# A sensitive LC–MS/MS method for isomer separation and quantitative determination of 51 pyrrolizidine alkaloids and two tropane alkaloids in cow’s milk

**DOI:** 10.1007/s00216-022-04344-5

**Published:** 2022-10-01

**Authors:** Lisa Monika Klein, Angelika Miriam Gabler, Michael Rychlik, Christoph Gottschalk, Florian Kaltner

**Affiliations:** 1 Chair of Food Safety and Analytics, Faculty of Veterinary Medicine, Ludwig Maximilian University of Munich, Schoenleutnerstr. 8, 85764 Oberschleissheim, Germany; 2 Chair of Analytical Food Chemistry, TUM School of Life Sciences Weihenstephan, Technical University of Munich, Maximus-von-Imhof-Forum 2, 85354 Freising, Germany; 3 Present Address: Unit Plant Toxins and Mycotoxins, Department Safety in the Food Chain, German Federal Institute for Risk Assessment, Max-Dohrn-Str. 8-10, 10589 Berlin, Germany; 4 Present Address: Institute of Food Chemistry and Food Biotechnology, Justus Liebig University of Giessen, Heinrich-Buff-Ring 17-19, 35392 Giessen, Germany

**Keywords:** Pyrrolizidine alkaloids, Tropane alkaloids, Milk, Method development, Liquid chromatography tandem mass spectrometry

## Abstract

1,2-Unsaturated pyrrolizidine alkaloids (PA), their corresponding *N*-oxides (PANO), and tropane alkaloids (TA) are toxic secondary plant metabolites. Their possible transfer into the milk of dairy cows has been studied in feeding trials; however, only few data on the occurrence of these toxins in milk are available. In this study, the development of a sensitive analytical approach for the simultaneous detection and quantification of a broad range of 54 PA/PANO as well as of the TA atropine and scopolamine in milk of dairy cows is presented. The method optimisation focused on sensitivity and separation of PA/PANO isomers. Milk samples were extracted using liquid–liquid extraction with aqueous formic acid and n-hexane, followed by a cation-exchange solid-phase extraction for purification. Reversed phase liquid chromatography tandem mass spectrometry (LC–MS/MS) analysis was performed using alkaline solvent conditions. Validation proved low limits of detection and quantification of 0.005 to 0.054 µg/L and of 0.009 to 0.123 µg/L, respectively. For 51 of the 54 tested PA/PANO and both TA, the recovery rates ranged from 64 to 127% with repeatability (RSD_r_) values below 15% at concentration levels of 0.05 and 0.50 µg/L and below 8% at a concentration level of 3.00 µg/L. Only three PANO did not match the validation criteria and were therefore regarded as semiquantitative. The final method was applied to 15 milk samples obtained from milk vending stations at farms and from local marketers in Bavaria, Germany. In three of the milk samples, traces of PA were detected.

**Graphical abstract:**

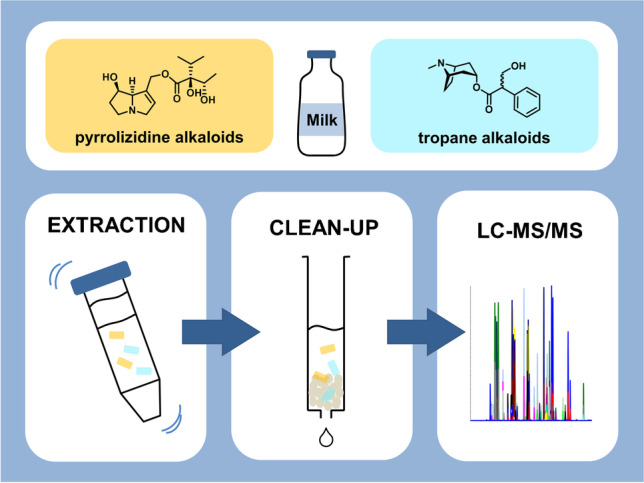

**Supplementary Information:**

The online version contains supplementary material available at 10.1007/s00216-022-04344-5.

## Introduction

**Fig. 1:**
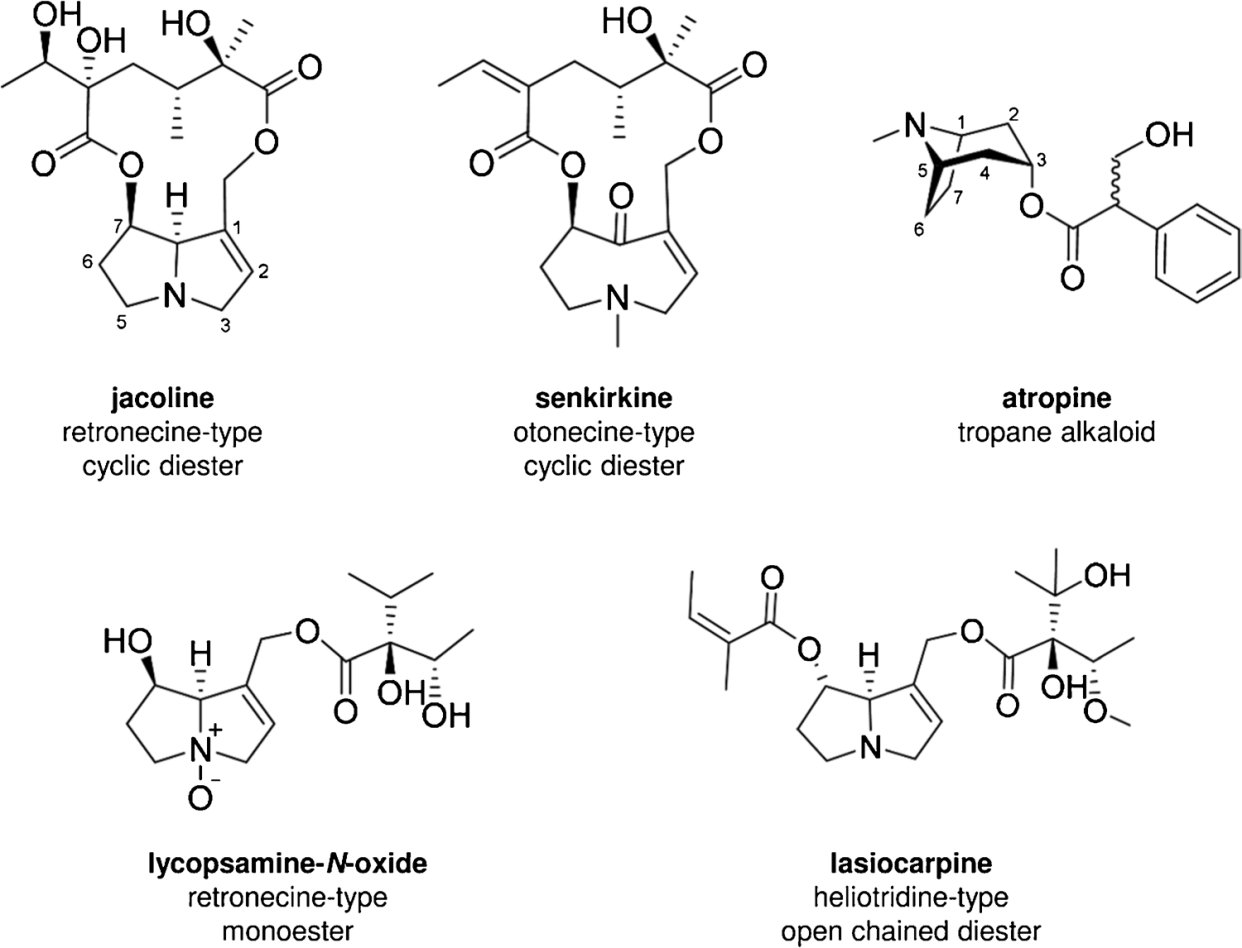
Examples of pyrrolizidine alkaloids, their corresponding *N*-oxides and tropane alkaloids, representing different core structures and grades of esterification

Pyrrolizidine alkaloids (PA) and tropane alkaloids (TA) are secondary plant metabolites. It is estimated that pyrrolizidine alkaloids and their corresponding *N*-oxides (PANO) are synthesised by more than 6,000 plants to protect them against herbivores [1, 2]. The main PA/PANO-producing plants are members of the families Asteraceae, Boraginaceae, and Leguminosae [2]. To this day, more than 660 different PA/PANO are known, with many of them exhibiting genotoxic and carcinogenic effects on humans and livestock [3, 4]. All PA/PANO share a common 1-hydroxymethyl-7-hydroxypyrrolizidine core structure (necine base) while a double bond at the 1,2-position is crucial for their toxic potential [5]. 1,2-Unsaturated PA/PANO can be of the retronecine, heliotridine, or otonecine type. These necine bases occur mostly as mono-, di-, or cyclic diesters with the necine base esterified at the *C*7- and/or at the *C*9-atom with mono- or dicarboxylic acids (necine acids) (Fig. 1) [4].

PA/PANO are categorised as protoxins as they reveal their toxic potential solely after metabolic activation by hepatic cytochrome P450 monooxygenases within the phase I metabolism. The highly reactive pyrrole esters formed in the liver bind irreversibly to DNA or proteins thereby inducing toxic effects [6]. Acute poisoning with PA/PANO leads to pathognomonic hepatic sinusoidal obstruction syndrome (HSOS, or veno-occlusive disease, VOD) [7]. A long-term intake even of low doses of PA/PANO is related to liver cirrhosis, liver and kidney cancer, and adverse effects on the lung (pulmonary arterial hypertension, PAH) and other organs.

Plants producing TA belong predominantly to the plant families Solanaceae, Brassicaceae, Erythroxylaceae, Proteaceae, Euphorbiaceae, Rhizophoraceae, and Convolvulaceae [8]. The common structural element of TA is the (1R,5S)-8-methyl-8-azabicyclo[3.2.1]octane core structure (tropane). In particular, TA produced by plants of the family Solanaceae are composed of tropine (3α-hydroxy-tropane) esterified to a carboxylic acid [9, 10]. Until now, more than 200 TA are known but (-)-hyoscyamine (pharmaceutical active isomer of the racemic mixture atropine) and (-)-scopolamine produced by plants of the Solanaceae family are the best-studied TA occurring in food and feed [11]. The toxicological effects of (-)-hyoscyamine and (-)-scopolamine are induced by their non-specific binding characteristic to muscarinic acetylcholine receptors of the central and autonomous nervous system. Clinical symptoms of TA intoxication include among others decreased production of secretions, visual impairment due to pupillary dilation and paralysis of accommodation, reduction in gastrointestinal tone, changes in heart rate, hallucinations, and respiratory depression. Based on the current information, in contrast to PA/PANO, TA do not exhibit genotoxic or chronic toxicity [12].

With regard to their occurrence and toxic properties, Commission Regulation (EC) No. 1881/2006 was amended by Commission Regulation (EU) No. 2020/2040 to set regulatory limits for PA/PANO content in tea, herbal infusions, spices, and other plant-based foodstuffs [13, 14]. These are considered to be the highest contaminated food commodities. Like for PA/PANO, plant-based foodstuff is considered to be the main source of TA [12]. In particular, cereals, cereal-based food, and several seeds are at risk to be contaminated with seeds of *Datura stramonium* L. As a consequence, with the Commission Regulation (EU) No. 2021/1408, maximum levels of atropine and scopolamine in herbal tea and in cereal-based foods which contain millet, sorghum, buckwheat, maize, or their derived products were established [15].

Plants containing PA/PANO and TA can also grow on farm grassland which can then lead to a contamination of feed. So far, feed regulations only cover the occurrence of plant material of alkaloid-containing species such as *Crotalaria* spp. and *Datura stramonium* L. as harmful botanical impurities, but not the toxic alkaloids themselves (2002/32/EC) [16]. PA/PANO contamination of milk after supplementation with PA/PANO-containing plant material was first proven in an animal study with cows by Dickinson et al. in 1976 [17]. The carry-over of PA/PANO from plant material into the milk of dairy cows was estimated to range between 0.01 and 0.1%, depending on the study setup and the plant species used [18, 19]. It was also found that individual PA differ in their carry-over rate. For ragwort, jacoline was found to be the main PA/PANO detected in the milk of dairy cows, although being only a minor component of the initial plant material [18, 19]. Regarding TA, the transfer of atropine and scopolamine from feed to raw milk of dairy cows was recently described, with transfer rates of 0.037 and 0.007%, respectively [20].

Only few studies investigated the occurrence of PA/PANO in retail milk and milk products [21–24]. Huybrechts and Callebaut investigated the occurrence of PA/PANO in 63 retail milk samples from Belgium using a liquid chromatography tandem mass spectrometry (LC–MS/MS) method including 10 PA and 6 PANO with a limit of quantification (LOQ) of 0.003 to 0.033 µg/kg [24]. In eight of these milk samples, PA/PANO were found up to a total content of 0.061 µg/kg. A larger study analysed 182 milk samples from several European countries for 19 PA and 16 PANO with a LOQ of 0.1 µg/L. PA/PANO concentrations up to 0.17 µg/L were detected in eleven of the milk samples [21]. In another study conducted by Chung and Lam, a method including 15 PA and 13 PANO and with the LOQ ranging from 0.010 to 0.087 µg/kg was used [23]. PA/PANO were detected in none of the six whole milk samples. Overall, little is known about the occurrence of PA/PANO and TA in retail milk or raw milk purchased directly from dairy farms. To the best of our knowledge, so far, only Zheng et al. investigated the occurrence of atropine and scopolamine in ten retail milks using a method which achieved LOQ ranging from 2.0 to 5.0 µg/kg [25].

Mainly, LC–MS/MS combined with liquid–liquid extraction (LLE), ultracentrifugation and/or freeze-out followed by solid-phase extraction (SPE) is used for PA/PANO analysis in milk (detailed information in Supplementary Material (ESM) Table S1). This results in low limits of detection (LOD) and LOQ which are of special importance as relatively low amounts of PA/PANO were found in milk so far. Another approach using direct analysis in real-time mass spectrometry was also developed but resulted in LOD ranging from 0.55 to 0.85 µg/L [26]. As there are still barely data on typical PA/PANO profiles in milk, it is also of high relevance to cover as many PA/PANO as possible. Hence, multi-analyte methods should be applied for analysis. Current LC–MS/MS methods for PA/PANO or TA analysis in several foods have been reviewed recently by Casado et al. and González-Gómez et al. [27, 28].

Currently, only few analytical methods for the analysis of PA/PANO or TA in milk are published (ESM Table S1). Thus, new sensitive and reliable analytical methods are important to allow further investigation into this highly consumed food commodity. To the best of our knowledge, no analytical method allowing the sensitive and simultaneous analysis of PA/PANO and TA in milk is available yet. To address this issue, an in-house validated cation-exchange SPE method combined with a sensitive LC–MS/MS method for the quantification of 30 1,2-unsaturated PA and 24 1,2-unsaturated PANO as well as atropine and scopolamine in raw milk of dairy cow is presented in this study.

## Materials and methods

### Chemical reagents and standards

The compounds 7-*O*-acetylintermedine, 7-*O*-acetylintermedine-*N*-oxide, 7-*O*-acetyllycopsamine, atropine, echimidine, echimidine-*N*-oxide, erucifoline, erucifoline-*N*-oxide, europine, europine-*N*-oxide, heliotrine, heliotrine-*N*-oxide, intermedine, intermedine-*N*-oxide, jacobine, jacobine-*N*-oxide, lasiocarpine, lasiocarpine-*N*-oxide, lycopsamine, lycopsamine-*N*-oxide, monocrotaline, monocrotaline-*N*-oxide, retrorsine, retrorsine-*N*-oxide, (-)-scopolamine, senecionine, senecionine-*N*-oxide, seneciphylline, seneciphylline-*N*-oxide, senecivernine, senecivernine-*N*-oxide, senkirkine, and trichodesmine were obtained from PhytoLab (Vestenbergsgreuth, Germany). 7-*O*-Acetyllycopsamine-*N*-oxide, echinatine, echinatine-*N*-oxide, heliosupine, heliosupine-*N*-oxide, indicine, indicine-*N*-oxide, integerrimine, integerrimine-*N*-oxide, jacoline, jacoline-*N*-oxide, jaconine, merenskine, merenskine-*N*-oxide, merepoxine, merepoxine-*N*-oxide, riddelliine, riddelliine-*N*-oxide, rinderine, rinderine-*N*-oxide, spartioidine, sceleratine, sceleratine-*N*-oxide, usaramine, and usaramine-*N*-oxide were purchased from CFM Oskar Tropitzsch (Marktredwitz, Germany).

Stock solutions (*c* = 1 mg/mL) of 7-*O*-acetyllycopsamine, 7-*O*-acetylintermedine, 7-*O*-acetyllycopsamine-*N*-oxide, 7-*O*-acetylintermedine-*N*-oxide, merepoxine, sceleratine, and sceleratine-*N*-oxide were prepared with acetonitrile/water (50/50, v/v) and with pure acetonitrile in case of indicine and indicine-*N*-oxide. All other substances were dissolved in methanol to prepare stock solutions (*c* = 1 mg/mL) of each PA/PANO and TA. Aliquots of the stock solutions were diluted in methanol to prepare a mix solution containing all substances and a mix solution containing all substances except 7-*O*-acetylintermedine-*N*-oxide and 7-*O*-acetyllycopsamine-*N*-oxide (*c* = 10 µg/mL of each PA/PANO/TA). Seven mix solutions with isomeric compounds distributed among different solutions (*c* = 1 µg/mL of each included substance) were prepared in the same way. The stock solutions and the mix solutions were stored at − 20 °C in the dark. For all experiments, analytical grade n-hexane, analytical grade 25% ammonia solution, LC–MS-grade methanol, and LC–MS-grade acetonitrile, acquired from Th. Geyer (Renningen, Germany), were used. Ultrapure water was obtained by water purification through an UltraClear™ TP UV UF TM from Evoqua Water Technologies (Barsbüttel, Germany). Formic acid was purchased from Merck (Darmstadt, Germany). Ammonium carbonate (HPLC-grade) and ammonium formate (LC–MS-grade) used as additive for LC–MS/MS solvents were obtained from Fisher Scientific (Schwerte, Germany) and Honeywell (Seelze, Germany), respectively.

### Instrumentation

For measurements, a Shimadzu high-performance liquid chromatography (HPLC) apparatus (DGU-405, LC-20AB, SIL-20AC HT, CTO-20AC, CBM-20A, Duisburg, Germany) coupled to an API4000 triple quadrupole MS (Sciex, Darmstadt, Germany) was used. The following MS ion source parameters were applied for electrospray ionisation (ESI): ionisation voltage, 2500 V; nebuliser gas, 50 psi; heating gas, 50 psi; curtain gas, 30 psi; temperature, 600 °C; collision gas level, 7. Data acquisition and processing were carried out with Analyst (version 1.6.2, Sciex) and MultiQuant software (version 3.0.1, Sciex). Processed data were further analysed using Microsoft Excel (version 2019, Microsoft) and OriginPro (version 2020, Origin Lab). Chemical structures were drawn with ChemDraw software (version 20.1.1, PerkinElmer) and chromatograms were exported from Analyst software. Figures were labelled with PowerPoint (version 2019, Microsoft).

### Samples

For method development and validation, raw milk was purchased at two different dates directly from a farm with a milk vending station (District Freising, Bavaria, Germany). For demonstrating the method’s applicability, raw milks (*n* = 10) were obtained at other farms with milk vending stations in Bavaria. Additionally, pasteurised milks (*n* = 5) were purchased from a regional marketer (District Munich, Bavaria, Germany) and a self-service machine (District Oberallgäu, Bavaria, Germany). Detailed information on the milk samples is available in the ESM Table S2. Each milk sample was stored in aliquots at − 20 °C until extraction.

### Development of the LC–MS/MS method

For the determination of multiple reaction monitoring (MRM) transitions, single substance solutions (*c* = 100 ng/mL) in methanol/water (50/50, v/v, containing 5 mmol/L ammonium formate) were directly injected into the MS via a syringe pump (Harvard Apparatus, Holliston, USA). The compound optimisation was performed using Analyst software. Single substance solutions were measured using a previously published LC–MS/MS method from Kaltner et al. to assess the signal intensity and the signal-to-noise ratio (S/N) of the eight selected mass transitions [29]. Two MRM transitions per substance, all [M + H]^+^ in positive electrospray ionisation (ESI +) mode, were selected for further method development.

The initial HPLC method development was conducted with a 150 × 2.1 mm Kinetex™ 5 µm EVO C18 column (Phenomenex, Aschaffenburg, Germany). A 100 × 2.1 mm Kinetex™ 2.6 µm EVO C18 column was tested with the chosen mobile phases to further improve the performance of the HPLC method (Phenomenex, Aschaffenburg, Germany). Each LC-column was protected by a SecurityGuard™ ULTRA EVO C18 2.1 mm pre-column (Phenomenex, Aschaffenburg, Germany). For the Kinetex™ 5 µm EVO C18 column, a flow rate of 0.4 mL/min and an injection volume of 20 µL was used. The Kinetex™ 2.6 µm EVO C18 column was operated at a flow rate of 0.3 mL/min and an injection volume of 10 µL. The oven temperature was kept at 30 °C during the whole method development.

The 150 × 2.1 mm Kinetex™ 5 µm EVO C18 column was tested in combination with several gradients under acidic and alkaline conditions, using two standard mix solutions containing all substances (*c* = 25 ng/mL, 5 ng/mL). Seven additional solutions (*c* = 25 ng/mL), with isomeric analytes distributed among different solutions, were used to confirm the separation and the assignment to the compounds. For separation under acidic conditions, ammonium formate and formic acid were added to water (solvent A) and acetonitrile/water (95/5, v/v, solvent B) to final concentrations of 5 mmol/L and 26.5 mmol/L (= 0.1 vol.%), respectively, as described by Kaltner et al. [29]. In case of alkaline conditions, solvent A contained 10 mmoL/L ammonium carbonate in water and acetonitrile was used as solvent B as described by Chen et al. [32]. The solvent mixture used to dilute the stock solutions to the final concentration of the standards was either mobile phase A or methanol/water (10/90, v/v). Selected HPLC gradients were combined with the application of scheduled MRM (sMRM) detection windows of 120 s set in the MS/MS method.

The chromatographic resolution (Eq. 1) was calculated to quantitatively assess the quality of the separation of critical isomers.

1 $${R}_{\mathrm{s}}= \frac{2({t}_{\mathrm{R}2}-{t}_{R1})}{{w}_{\mathrm{B}1}+{w}_{\mathrm{B}2}}$$

*t*_R1/2_: retention time of peaks [min]
*w*_B1/2_: baseline peak width of peaks [min]

Additionally, for atropine, the tailing factor (Eq. 2) was calculated as follows.

2 $$T= \frac{{w}_{\mathrm{x}}}{2f}$$

*w*_x_: width of the peak determined at 5% from the baseline of the peak height
*f*: distance between peak maximum and peak front at 5% from the baseline of the peak height

Differences in sensitivity were evaluated for non-interfering analytes by comparing S/N and mean signal area of two injections of a 5 ng/mL standard mix of all substances in methanol/water (10/90, v/v).

### Development of sample extraction and clean-up

Extraction and clean-up procedures using C18 or polymeric cation exchange (PCX) cartridges were tested. Furthermore, using a protocol with PCX material, the impact of a LLE using n-hexane was evaluated. For all experiments, the frozen milk samples were thawed in a water bath at 30 °C and homogenised by shaking. Sample extraction and clean-up procedures were conducted in four replicates each. Aliquots of 3 mL milk were spiked to reach a concentration of 12.3 ng/mL in the final measuring solution.

A slightly adapted version of the extraction and clean-up procedures protocol of Mulder et al. using C18 cartridges was conducted [21]. In detail, 3 mL of milk was extracted with 30 mL of 0.2% formic acid and 15 mL n-hexane in a 50 mL centrifuge tube on a horizontal shaker (GFL, Burgwedel, Germany) for 30 min at 450 rpm and centrifuged at 2600 × *g* and 20 °C for 15 min. A 25-mL aliquot of the aqueous phase was adjusted to a pH of 9–10 using 25% ammonia and pH test strips (Th. Geyer, Renningen, Germany) and centrifuged again at 3600 × *g* and 20 °C for 15 min. SPE clean-up was conducted with a Strata-X C18 6 mL 200 mg (Phenomenex, Aschaffenburg, Germany) conditioned with 6 mL of methanol followed by 6 mL of 0.1% ammonia solution. After applying 15 mL of the aqueous raw extract, the column was washed with 6 mL 0.1% ammonia solution and dried under vacuum before elution with 6 mL of methanol. The eluates were evaporated to dryness under a gentle stream of nitrogen at 50 °C. The residues were reconstituted in 500 µL methanol/water (10/90, v/v), ultrasonicated, vortexed for 30 s, and filtered through a 0.2 µm polyvinylidene difluoride (PVDF) syringe filter (Macherey–Nagel, Düren, Germany).

For extraction and clean-up with PCX cartridges, 30 mL of 2% formic acid or 30 mL of 2% formic acid and 15 mL of n-hexane were added to 3 mL of milk samples. The centrifuge tubes were placed on a horizontal shaker for 30 min at 450 rpm. Samples with added n-hexane were centrifuged at 2600 × *g* and 20 °C for 15 min. Afterwards, 25 mL of the aqueous phase was transferred into a new 50 mL centrifuge tube and centrifuged again at 3600 × *g* and 20 °C for 15 min. Samples containing no n-hexane were centrifuged directly at 3600 × *g* and 20 °C for 15 min before filtered through a folded filter paper (Whatman, London, UK). For PCX SPE, the procedure suggested by Kaltner et al. for plant-based matrices was tested [29]. Therefore, Bond Elut Plexa PCX 6 mL 500 mg cartridges (Agilent, Waldbronn, Germany) were conditioned with 5 mL of methanol and 5 mL of 2% formic acid before loading 15 mL of the aqueous raw extract. After washing with 10 mL of water and 10 mL of methanol, PA/PANO/TA were eluted with 10 mL of ammoniated methanol (5%). The eluates were evaporated to dryness under a gentle stream of nitrogen at 50 °C. The residues were reconstituted in 1000 µL methanol/water (10/90, v/v), ultrasonicated, vortexed for 30 s, and filtered through a 0.2 µm PVDF syringe filter.

For estimation of the recovery and matrix suppression or enhancement, samples were analysed together with calibration standards ranging from 0.5 to 25 ng/mL in methanol/water (10/90, v/v) and the relative standard deviation (RSD) values were calculated. Additionally, the mass transition signals in the spiked and blank samples with and without further addition of n-hexane during extraction were visually inspected for changes in the baseline noise level.

Furthermore, Bond Elut Plexa PCX 6 mL 200 mg cartridges (Agilent, Waldbronn, Germany) and a volume of 500 µL methanol/water (10/90, v/v) for reconstitution were tested using the same PCX SPE procedure.

### Method validation

For in-house method validation, LOD and LOQ, recovery rates, linearity, repeatability, and matrix effects were evaluated. LOD and LOQ were determined according to the calibration curve procedure of German standard DIN 32,645 with a 1% error probability and an uncertainty of 33.3% [30]. Accordingly, twelve equidistant concentration levels ranging from 0.01 to 0.12 ng/mL and eleven equidistant concentration levels ranging from 0.2 to 1.2 ng/mL in blank milk extract were prepared. The milk extracts were prepared according to the final extraction and clean-up protocol; solely the reconstitution of the dried eluates was performed using 50 µL of the corresponding tenfold concentrated mix solutions of all 56 PA/PANO/TA in methanol and 450 µL water instead of 500 µL methanol/water (10/90, v/v). The calculated LOD for each analyte corresponds to the LOD of the less sensitive mass transition and the LOQ was the one of the more sensitive quantifier mass transition. Recovery experiments were performed using milk artificially contaminated at 0.05, 0.50, and 3.00 µg/L with five replicates each. The same data set was used to calculate the repeatability (RSD_r_) of the method. The linearity of the calibration curve was evaluated according to SANTE/12682/2019 and by using the Mandel’s *F*-test [31]. The correlation coefficients were calculated using Microsoft Excel 2019. Enhancement or suppression effects on the ion signal intensity were calculated based on the slopes of the regression curves (Eq. 3).

Matrix enhancement/suppression

3 $$SE= (1- \frac{{m}_{\mathrm{milk}}}{{m}_{\mathrm{solvent}}})\bullet 100\%$$

*m*_milk_: slope of the matrix-matched calibration curve
*m*_solvent_: slope of the solvent calibration curve

### Quality control and quantification

External matrix-matched calibration standards were freshly prepared each day. To obtain standards of 0, 0.1, 0.5, 1.25, 2.5, 5, and 10 ng/mL in milk extract, raw cow’s milk was prepared following the extraction and clean-up protocol and residues were reconditioned in 50 µL of a corresponding tenfold concentrated PA/PANO/TA standard mix solution in methanol and 450 µL water. All samples were analysed in duplicate. LC–MS/MS system’s stability was proven by injecting the calibration standards before, in the middle and after a sequence of sample runs. Linear regression curves including all standard injections and the point of origin were calculated using MultiQuant Software. The back-calculated concentrations of the single calibration points were checked for accuracy (80 <  *x*  < 120%) as described in SANTE/12682/2019. A PA/PANO/TA compound was confirmed in a sample if peaks of both sample replicates matched the respective retention time (± 0.10 min) and the ion ratio (± 20%) of quantifier and qualifier mass transitions obtained in the calibration standards. Results were not corrected for recovery rates. To calculate a PA/PANO sum content, individual PA/PANO, which were detected with a calculated concentration between the LOD and LOQ, were set as 0.5 times the LOQ. The results obtained from the tested milk samples were further confirmed by analysing blank milk spiked to the calculated concentrations of the detected analytes in triplicate.

## Results and discussion

In this method development, all 56 at that time commercially available 1,2-unsaturated PA/PANO and the TA atropine and scopolamine were included.

### Development of the LC–MS/MS method

The ESI + mode enabled measurement of all substances after compound optimisation of single PA/PANO/TA standard solutions. The finally optimised and selected mass transitions for PA/PANO/TA are summarised in Table 1.

**Table 1:** Retention times, mass transitions, and compound-specific voltage settings of the tandem mass spectrometer API 4000 (AB Sciex) for determination of 30 pyrrolizidine alkaloids, 26 pyrrolizidine alkaloid *N*-oxides and two tropane alkaloids in the developed LC–MS/MS method

Pyrrolizidine alkaloid/pyrrolizidine alkaloid *N*-oxide/tropane alkaloid	Abbrev	Retention time^a^ [min]	Precursor ion [*m*/*z*]	Product ions [*m*/*z*] (QN/QL)	DP [V]	CE [eV] (QN/QL)	CXP [V] (QN/QL)	Ion ratio^b^ (QL/QN)
Monocrotaline-*N*-oxide	McN	3.9	342.2	137.1 / 118.1	116	41 / 67	24 / 22	0.38
Jacoline-*N*-oxide	JlN	3.9	386.3	120.1 / 172.1	111	59 / 51	22 / 30	0.39
Sceleratine-*N*-oxide	SlN	4.2	386.3	118.1 / 340.2	121	51 /41	22 / 20	0.37
Intermedine-*N*-oxide	ImN	4.8	316.2	172.0 / 94.1	96	39 / 59	32 / 16	0.64
Indicine-*N*-oxide	IcN	5.0	316.2	172.1 / 94.1	101	39 / 61	30 / 16	0.64
Lycopsamine-*N*-oxide	LyN	5.0	316.2	172.1 / 138.1	86	39 / 39	30 / 24	0.66
Erucifoline-*N*-oxide	EcN	5.1	366.2	118.0 / 136.0	106	47 / 45	20 / 24	0.85
Europine-*N*-oxide	EuN	5.2	346.2	172.0 / 270.1	91	45 / 35	30 / 14	0.26
Echinatine-*N*-oxide	EnN	5.5	316.2	172.1 / 80.0	101	41 / 83	10 / 14	0.40
Rinderine-*N*-oxide	RrN	5.5	316.2	172.3 / 80.0	76	41 / 89	10 / 14	0.41
Jacobine-*N*-oxide	JbN	6.4	368.2	296.2 / 120.1	101	35 / 53	16 / 22	0.76
Riddelliine-*N*-oxide	RlN	6.5	366.1	93.8 / 118.2	96	65 / 51	10 / 6	0.70
Merepoxine-*N*-oxide	MxN	7.4	368.2	136.0 / 120.1	121	45 / 55	24 / 22	0.20
Merenskine-*N*-oxide	MkN	7.4	404.1	118.1 / 138.1	71	61 / 53	20 / 26	0.53
Usaramine-*N*-oxide	UsN	8.0	368.1	118.3 / 94.1	101	47 / 67	6 / 16	1.81
Heliotrine-*N*-oxide	HtN	8.2	330.2	172.2 / 111.0	96	39 / 59	30 / 20	0.35
Retrorsine-*N*-oxide	RsN	8.2	368.2	118.0 / 340.2	111	43 / 39	20 / 20	0.23
Jacoline	Jl	8.3	370.2	120.1 / 326.2	111	47 / 37	22 / 18	0.41
Intermedine	Im	8.5	300.1	94.0 / 138.0	91	39 / 29	16 / 24	1.18
Indicine	Ic	8.5	300.2	138.0 / 156.1	91	29 / 41	24 / 28	0.71
Lycopsamine	Ly	8.7	300.2	94.0 / 138.1	101	39 / 29	16 / 24	0.70
Monocrotaline	Mc	8.9	326.2	120.1 / 237.2	106	51 / 35	22 / 12	0.37
Seneciphylline-*N*-oxide	SpN	9.2	350.2	94.1 / 322.3	96	67 / 35	16 / 18	0.18
Sceleratine	Sl	9.2	370.2	138.1 / 342.2	116	43 / 39	24 / 18	0.23
7-*O*-Acetylintermedine-*N*-oxide	AcImN	9.5	358.2	214.1 / 180.1	91	39 / 41	38 / 32	0.54
7-*O*-Acetyllycopsamine-*N*-oxide	AcLyN	9.6	358.2	214.1 / 180.1	96	39 / 41	38 / 32	0.64
Europine	Eu	10.6	330.2	138.1 / 254.1	86	33 / 27	24 / 14	0.37
Integerrimine-*N*-oxide	IgN	10.9	352.2	118.0 / 136.0	101	43 / 47	20 / 24	0.75
Senecionine-*N*-oxide	ScN	11.2	352.1	118.0 / 324.2	106	45 / 37	20 / 18	0.22
Echinatine	En	11.2	300.1	138.2 / 94.1	81	31 / 57	8 / 16	0.29
Senecivernine-*N*-oxide	SvN	11.3	352.2	118.1 / 324.3	96	45 / 37	22 / 18	0.34
Rinderine	Rr	11.4	300.1	138.0 / 156.0	76	31 / 39	8 / 8	0.45
Senkirkine	Sk	11.6	366.3	168.2 / 150.1	96	43 / 39	8 / 26	0.41
Heliosupine-*N*-oxide	HsN	12.6	414.3	94.1 / 118.1	81	65 / 85	18 / 22	0.64
Erucifoline	Ec	13.2	350.2	120.0 / 138.1	96	41 / 41	22 / 24	0.67
Echimidine-*N*-oxide	EmN	13.2	414.3	254.3 / 352.2	91	43 / 35	14 / 20	0.36
Jacobine	Jb	13.7	352.2	120.0 / 280.2	111	43 / 33	20 / 16	0.96
Riddelliine	Rl	14.0	350.1	120.1 / 94.1	101	39 / 59	22 / 18	0.61
Heliotrine	Ht	14.5	314.2	138.0 / 156.0	76	29 / 39	24 / 28	0.29
Merepoxine	Mx	14.8	352.1	120.1 / 324.2	141	45 / 39	22 / 32	0.40
Lasiocarpine-*N*-oxide	LcN	15.1	428.3	254.1 / 352.3	91	41 / 35	14 / 20	0.60
Usaramine	Us	15.1	352.1	120.1 / 94.1	106	43 / 57	6 / 16	0.50
Atropine	At	15.1	290.2	124.1 / 93.1	86	35 / 43	6/ 16	0.50
Retrorsine	Rs	15.5	352.2	120.1 / 324.2	101	43 / 39	20 / 18	0.68
Trichodesmine	Td	15.6	354.2	222.2 / 308.2	86	41 / 30	12 / 15	0.13
Scopolamine	Sco	15.6	304.1	138.1 / 156.1	71	35 / 23	8 / 8	0.51
Jaconine	Jn	15.9	388.1	94.2 / 156.2	111	61 / 55	18 / 8	0.74
7-*O*-Acetylintermedine	AcIm	16.1	342.2	120.0 / 180.1	81	37 / 25	22 / 32	0.35
7-*O*-Acetyllycopsamine	AcLy	16.3	342.2	120.0 / 180.1	81	35 / 25	22 / 32	0.22
Spartioidine	Sd	16.4	334.1	119.9 / 94.1	101	39 / 53	6 / 18	0.60
Merenskine	Mk	16.5	388.2	120.0 / 138.0	121	51 / 45	22 / 24	0.86
Seneciphylline	Sp	16.9	334.2	120.1 / 306.2	111	39 / 35	22 / 18	0.71
Integerrimine	Ig	18.3	336.3	120.1 / 308.2	96	41 / 37	22 / 18	0.47
Senecionine	Sc	18.8	336.3	120.1 / 308.2	106	41 / 37	22 / 18	0.57
Senecivernine	Sv	19.3	336.2	120.1 / 308.2	96	43 / 39	22 / 18	0.76
Heliosupine	Hs	19.3	398.2	119.8 / 220.3	81	39 / 27	6 / 12	0.53
Echimidine	Em	19.8	398.3	120.0 / 220.1	76	35 / 25	22 / 12	0.31
Lasiocarpine	Lc	22.4	412.3	120.0 / 220.2	86	39 / 27	22 / 12	0.44

Entrance potential (EP) = 10 V for all analytes

Abbreviations: *Abbrev*. abbreviation, *QN* quantifier, *QL* qualifier, *DP* declustering potential, *CE* collision energy, *CXP* cell exit potential

^a^Determined using the final HPLC conditions

^b^Mean of three injections of a 5 ng/mL standard mix solution

High structural similarity of PA/PANO caused a series of identical fragment ions ([M + H]^+^), e.g., fragments with a mass-to-charge-ratio (*m*/*z*) of 152, 138, 120, or 94. In case of isomeric compound groups, this led to several identical mass transitions. Therefore, HPLC resolution was the key issue for successful identification of individual compounds.

Chromatographic resolution is especially crucial for the differentiation of the isomeric monoesters intermedine, indicine, lycopsamine, rinderine, and echinatine (*m*/*z *300) and their *N*-oxides (*m*/*z *316) as well as the cyclic diesters integerrimine, senecionine, and senecivernine (*m*/*z *336) and their *N*-oxides (*m*/*z *352), which were all included in the method development. In literature, mainly LC–MS/MS methods in reversed phase (RP) mode using C18 columns combined with both acidic or alkaline solvent conditions were reported for PA/PANO/TA analysis, with the latter solvent conditions being less common [29–41]. Only few methods were published using pentafluorophenyl (PFP) or hydrophilic interaction liquid chromatography (HILIC) columns [34, 42, 43].

In this study, a 150 × 2.1 mm Kinetex™ 5 µm EVO C18 column was initially used for method development and combined with acidic or alkaline mobile phase conditions, as described by Kaltner et al. and by Chen et al. [29, 32]. For acidic conditions, several gradients were tested but an increase in run time with a flatter gradient than previously applied by Kaltner et al. did not lead to better resolution of isomer peaks (data not shown) [29]. Therefore, the gradient suggested by Kaltner et al. with a run time of 16.5 min was used for the acidic solvent conditions. In contrast, the gradient optimised for alkaline solvent conditions resulted in a final run time of 22 min. Due to the medium to higher polarity of individual PA/PANO, the created gradients had to start with high aqueous proportions in the mobile phase (2% solvent B) and a flat gradient.

Using acidic mobile phase conditions, PA were eluted faster from the column than their corresponding PANO, whereas the *N*-oxides were eluted first when an alkaline solvent A was used (ESM Fig. S1). Regardless of the gradient designs, better peak shape and resolution were observed under alkaline conditions for all selected PA/PANO compound groups (Table 2).

**Table 2:** Resolution for selected isomeric compounds obtained under acidic solvent conditions (solvent A: water, solvent B: acetonitrile/water (95/5, v/v), both containing 5 mmol/L ammonium formate and 26.5 mmol/L formic acid) and alkaline solvent conditions (solvent A: 10 mmol/L ammonium carbonate in water, solvent B: acetonitrile) on a 150 × 2.1 mm Kinetex™ 5 µm EVO C18 column and under alkaline solvent conditions on a 100 × 2.1 mm Kinetex™ 2.6 µm EVO C18 column using a 5 ng/mL standard of 30 pyrrolizidine alkaloids, 26 pyrrolizidine alkaloid *N*-oxides, and two tropane alkaloids in methanol/water (10/90, v/v)

Isomer group	Acidic conditions	Alkaline conditions	
	150 × 2.1 mm Kinetex™ 5 µm EVO C18	150 × 2.1 mm Kinetex™ 5 µm EVO C18	100 × 2.1 mm Kinetex™ 2.6 µm EVO C18
7-*O*-Acetylintermedine, 7-*O*-acetyllycopsamine	1.3	1.1	1.8
Echimidine, heliosupine	0.4	1.9	> 2
Echinatine, indicine, intermedine, lycopsamine, rinderine	0.9, 0.6	1.0, > 2, 0.6	0.4, 1.0, > 2, 1.5
Echinatine-*N*-oxide, indicine-*N*-oxide, intermedine-*N*-oxide, lycopsamine-*N*-oxide, rinderine-*N*-oxide	1.2, 1.5	1.1, > 2	1.5, > 2
Integerrimine, senecionine, senecivernine	0.7	1.9, > 2	> 2, > 2
Integerrimine-*N*-oxide, senecionine-*N*-oxide, senecivernine-*N*-oxide	0.8	1.6, 0.7	2.0, 1.1
Jacobine, merepoxine, usaramine, retrorsine	> 2, > 2	> 2, 0.8, > 2	> 2, > 2, > 2
Jacobine-*N*-oxide, merepoxine-*N*-oxide, usaramine-*N*-oxide, retrorsine-*N*-oxide	1.1, > 2	> 2, > 2, 1.2	> 2, > 2, > 2
Jacoline-*N*-oxide, sceleratine-*N*-oxide	0.8	1.7	> 2
Jaconine, merenskine	0	> 2	> 2

In particular, intermedine, indicine, lycopsamine, rinderine, and echinatine (*m*/*z *300) were eluted at acidic conditions in three unshaped peaks with insufficient resolution (*R*_S_ = 0.9 and 0.6). At alkaline conditions, these isomers were eluted in four peaks with a *R*_S_ of 1.0, > 2, and 0.6 (chromatograms are available in ESM Fig. S2). Alkaline solvent conditions also allowed baseline separation for integerrimine, senecionine, and senecivernine (*R*_S_ = 1.9 and > 2) and integerrimine-*N*-oxide and senecionine-*N*-oxide (*R*_S_ = 1.6). Senecionine-*N*-oxide and senecivernine-*N*-oxide could also be clearly resolved with a *R*_S_ of 0.7 (ESM Fig. S3). Furthermore, while jaconine and merenskine were co-eluting under acidic conditions, baseline resolution (*R*_S_ > 2) was achieved under alkaline solvent conditions as well (ESM Fig. S1). For atropine, peak tailing was observed under acidic conditions (*T* = 1.6) but even more under alkaline conditions (*T* = 2.2). This was assumed to be attributed to the pK_a_ of 9.43 for atropine.

Overall, using 10 mmol/L ammonium carbonate and acetonitrile as solvents consequently resulted in better S/N ratios. The alkaline solvent conditions led to more sensitive signals (up to 3.3-fold in case of riddelliine-*N*-oxide) compared to the tested acidic solvent conditions (data not shown).

In a second step, the dimensions of the column were altered to further improve the resolution of critical isomer pairs (Table 2). Using a 100 × 2.1 mm Kinetex™ 2.6 µm EVO C18 column instead of a 150 × 2.1 mm Kinetex™ 5 µm EVO C18 column allowed a reduction of the injection volume from 20 to 10 µL with a median decrease of peak height of only 35% for all 56 analytes in methanol/water (10/90, v/v) (data not shown). With regard to extracted milk samples, choosing an identical column with smaller particle size reduced the matrix load on the column which should be accompanied with reduced matrix suppression and increased shelf-life of the column. The gradient was further adjusted to a total run time of 24.0 min and 0% solvent B at 0.0 min with a steep increase to 5% solvent B within the first 12 s, similar to the start of the gradient applied by Chen et al. [32]. These changes improved the peak shape and therefore the sensitivity and separation for early eluting analytes like jacoline-*N*-oxide, sceleratine-*N*-oxide, and monocrotaline-*N*-oxide. It also resulted in a sufficient separation of echinatine and rinderine (*R*_S_ = 1.5) as well as intermedine-*N*-oxide and indicine-*N*-oxide/lycopsamine-*N*-oxide (*R*_S_ = 1.5). In addition, the differentiation between intermedine and indicine was possible (*R*_S_ = 0.4) but not sufficient for an individual quantification (ESM Fig. S2). A separation of the isomeric compound pairs indicine-*N*-oxide and lycopsamine-*N*-oxide as well as echinatine-*N*-oxide and rinderine-*N*-oxide was not possible at all. The peak tailing observed for atropine was also reduced to a *T* of 1.5 by using the 100 × 2.1 mm Kinetex™ 2.6 µm EVO C18 column. With the smaller particle size of the column, the flow rate had to be reduced to 0.3 mL/min in order to stay within the pressure limits of the HPLC apparatus. In consequence, an 18% decrease of solvent consumption was achieved and a re-equilibration time of 7.0 min was needed under the described conditions.

The presented method was capable to achieve good sensitivity and separation for most of the critical isomeric pairs out of a large set of 56 PA/PANO analytes on a standard HPLC system within a run time of 31 min (including re-equilibration). Recently published multi-PA/PANO methods mainly used ultra high-performance liquid chromatography (UHPLC) systems resulting in short run times of 11 to 25 min [32, 34, 37, 41, 44]. However, most of the methods published before did not include as many compounds from the isomeric group consisting of intermedine, indicine, lycopsamine, echinatine, and rinderine or their *N*-oxides, respectively. To the best of our knowledge, sufficient resolution of all five isomers was so far only reported in a study using a 2D-LC setup [45]. Therefore, some authors conclude to reanalyse samples with a complementary RP-HPLC approach under different pH conditions or a HILIC method if inseparable PA/PANO were detected with the initially used LC method [32, 34, 41]. While the isomer pairs rinderine-*N*-oxide and echinatine-*N*-oxide and indicine-*N*-oxide and lycopsamine-*N*-oxide remained inseparable under the presented alkaline solvent conditions, they were separated by the tested acidic method, however co-eluting with other isomers instead [29].

### Development of the sample extraction and clean-up

So far, only few different approaches for PA/PANO or for TA extraction and clean-up from milk or milk products using LC–MS/MS analysis were published (ESM Table S1). Commonly, either time-consuming freeze-out steps, ultracentrifugation, or LLE with n-hexane were applied to degrease milk samples. As PA/PANO and TA content was expected to be relatively low in milk and matrix components interfere in the LC–MS/MS analysis, a SPE clean-up with C18 or cation exchange material (SCX, PCX) and concentration steps are usually mandatory [21, 44, 46, 47, 48]. Methods with both C18 and cation exchange material were already successfully applied for simultaneous analysis of PA/PANO and TA in plant-based food [34, 49].

**Fig. 2:**
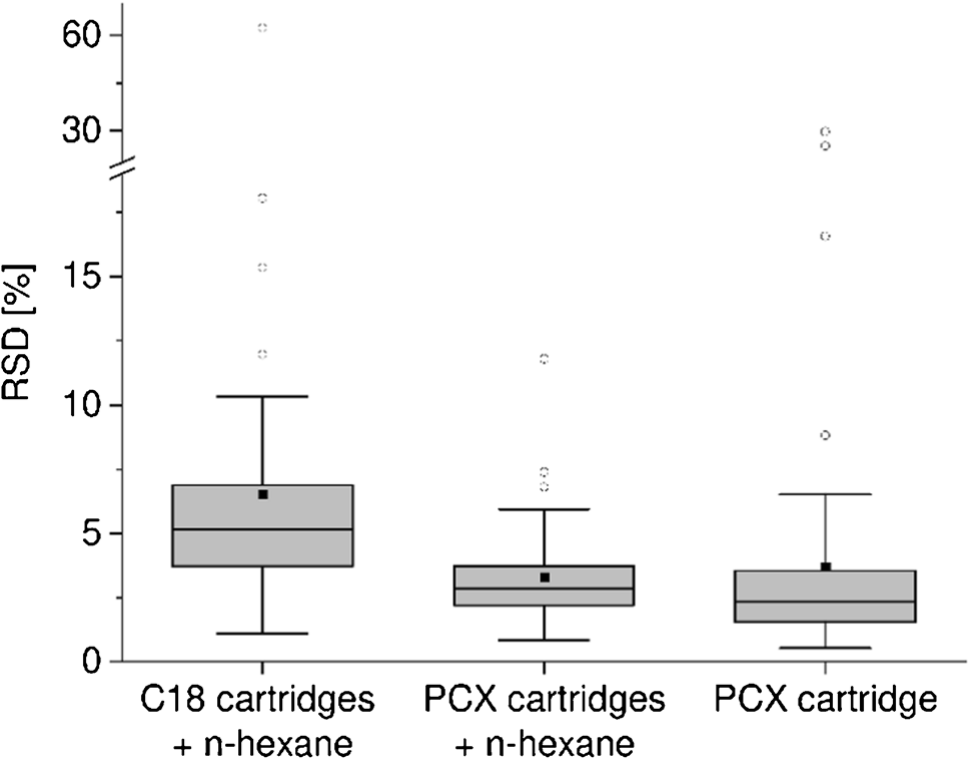
Box and whisker plot of the precision, expressed as the relative standard deviation (RSD), of extraction and clean-up procedures using C18 cartridges with n-hexane (*n* = 3) and polymer cation exchange (PCX) cartridges with and without n-hexane (*n* = 4). Replicates were spiked to a concentration of 12.3 ng/mL in the final measuring solution. The box corresponds to the range in which the middle 50% of the data are located. Whiskers mark the 1.5 interquartile range. Circles indicate individual outliers outside the 1.5 interquartile range. Squares represent the mean and lines the median

In this study, C18 SPE material and PCX SPE material were evaluated for sample clean-up. PCX cartridges showed easier handling as an acidic raw extract can be applied directly on the PCX cartridges, avoiding time-consuming and error-prone pH adjustment to neutral or alkaline pH conditions. The quantification of samples using calibration standards in methanol/water resulted in recoveries of 0.5 to 127% with a mean of 62% for the PCX cartridges and 7.4 to 100% with a mean of 53% for the C18 cartridges (detailed information in ESM Table S3). Considering that it was not corrected for matrix suppression, no difference in the overall recovery could be stated. Nevertheless, individual PA/PANO showed highly different results. 7-*O*-Acetylintermedine-*N*-oxide and 7-*O*-acetyllycopsamine-*N*-oxide showed very poor recovery results below 1% when PCX cartridges were used. This is in line with previous observations by Kaltner et al. [29]. Consequently, 7-*O*-acetylintermedine-*N*-oxide and 7-*O*-acetyllycopsamine-*N*-oxide were excluded for further method development with PCX cartridges. For merenskine-*N*-oxide and the late eluting compound lasiocarpine, poor recovery or extensive matrix suppression was observed for both SPE materials (ESM Table S3). Regarding the precision, the mean RSD was 3.3% when using the PCX clean-up protocol compared to a mean RSD of 6.5% using C18 cartridges for clean-up (Fig. 2 and detailed information in ESM Table S3).

Moreover, the maximum RSD values were 11.8% for PCX material and 62.4% for C18 material. In particular, the PA/PANO including a chlorine atom, namely jaconine, merenskine, and merenskine-*N*-oxide showed better precision when PCX material was used.

As PCX SPE material is based on ionic interactions between the analytes and the SPE material, fat molecules are unlikely to be retained on PCX cartridges due to their hydrophobicity. Based on this, the impact of the LLE with n-hexane was additionally assessed. For samples which were prepared without n-hexane, the recovery calculated using calibration standards in methanol/water (10/90, v/v) ranged between 0.6 and 106% with a mean of 59%. This was equal to the data acquired with n-hexane (ESM Table S3). Without the addition of n-hexane, the RSD ranged from 0.5 to 29.6% with a mean RSD value of 3.7%. Therefore, the addition of n-hexane to degrease the samples resulted in slightly improved precision, while both approaches showed very good mean RSD values below 4% (Fig. 2 and detailed information in ESM Table S3). Solely, for merenskine-*N*-oxide, a RSD value of 29.6% was observed. Differences were observed in the height of the baseline noise especially for typical PANO mass transitions such as 316.2 → 172.1 or 352.2 → 118.0. In conclusion, a clean-up solely with PCX cartridges was in general already sufficient for the analysis of PA/PANO and TA in milk, but due to less interfering noise signals and to protect the HPLC system from faster wear, a LLE with n-hexane was integrated in the extraction and clean-up procedure.

Clean-up with PCX cartridges was further optimised for raw cow milk. The use of Bond Elut Plexa PCX 6 mL 200 mg instead of Bond Elut Plexa PCX 6 mL 500 mg resulted in a considerable reduction in costs and time consumption. The washing procedure remained the same. In addition, the dried eluates were reconstituted in 500 µL instead of 1000 µL methanol/water (10/90, v/v) to achieve lower LOD and LOQ.

### Final method for the determination of PA/PANO and TA in milk

Three millilitres of the thawed sample was extracted with 30 mL 2% aqueous formic acid and 15 mL n-hexane in a 50 mL centrifuge tube at 450 rpm and room temperature in a horizontal shaker for 30 min. After centrifugation (2600 × *g*, 15 min, 20 °C), the aqueous phase was transferred to a new 50 mL centrifuge tube. SPE Bond Elut Plexa PCX 200 mg cartridges (Agilent, Waldbronn, Germany) were conditioned with 5 mL methanol and 5 mL 2% aqueous formic acid before loading 15 mL of the aqueous raw sample extract onto the cartridges. After washing with 10 mL 2% aqueous formic acid and 10 mL of methanol, the analytes were eluted with 6 mL of ammoniated methanol (5%) into a glass vial. The eluates were dried in a water bath at 50 °C under a gentle stream of nitrogen. The residue was reconstituted in 500 µL of methanol/water (10/90, v/v), vortexed, ultrasonicated for 30 s, and filtered into a glass vial using a syringe filter (0.2 µm, PVDF).

Chromatographic separation was performed with a 100 × 2.1 mm Kinetex™ 2.6 μm EVO C18 100 Å column protected by a SecurityGuard™ ULTRA EVO C18 2.1 mm pre-column (both Phenomenex, Aschaffenburg, Germany). Aqueous 10 mM ammonium carbonate at pH 9.0 (A) and acetonitrile (B) were used as solvents for HPLC separation. The binary gradient conditions were the following: 0.0 min 0% B, 0.2 min 5% B, 6.0 min 10% B, 19.0 min 28.6% B, 22.5 min 33,6% B, 22.6 min 95% B, and 24.0 min 95% B. The flow rate was 0.3 mL/min. The column was equilibrated at starting condition for 7.0 min prior to each run. The column oven temperature was set to 30 °C and injection volume was 10 µL.

### Method validation

The final method was validated using raw cow’s milk purchased from a milk vending station. For determination of recovery and repeatability, the milk was artificially contaminated to individual analyte concentrations of 0.05, 0.50, and 3.00 µg/L. These analyte concentrations represent amounts near the LOQ, tenfold the LOQ, and in the upper calibration range for most of the PA/PANO/TA. The validation results are displayed in Table 3.

**Table 3:** Overview of the validation data of the final method. Average recovery rates and repeatability (expressed as relative standard deviation, RSD_r_) at three spiking levels with *n* = 5 replicates each, limit of detection (LOD) and limit of quantification (LOQ), linearity (expressed as the coefficient of correlation, *R*^2^), and matrix suppression

Analyte^a^	Level 0.05 µg/L		Level 0.50 µg/L		Level 3.00 µg/L		LOD	LOQ	*R*^2^	Matrix
	Recovery [%]	RSD_r_ [%]	Recovery [%]	RSD_r_ [%]	Recovery [%]	RSD_r_ [%]	[µg/L]	[µg/L]	(LOD — 5.50 µg/L)	suppression^b^ [%]
AcIm	94.0	5.0	95.0	6.7	85.7	2.3	0.007	0.009	0.9984^c^	35.1
AcLy	92.3	4.2	95.6	6.5	82.7	3.1	0.007	0.020	0.9992^c^	33.0
At	87.0	6.5	96.2	7.8	92.9	2.2	0.010	0.021	0.9926	37.7
Ec	106.4	7.8	102.5	5.6	95.1	3.6	0.009	0.009	0.9958	52.9
EcN	104.3	7.2	97.0	2.7	85.0	3.5	0.012	0.036	0.9972	52.5
Em	103.3	3.4	102.1	7.6	87.4	2.9	0.011	0.026	0.9992^c^	43.1
EmN	99.2	5.1	98.4	5.9	90.1	4.5	0.008	0.018	0.9992^c^	33.6
En	114.4	3.2	100.2	4.3	91.3	2.3	0.007	0.019	0.9996^c^	38.0
EnN/RrN	102.6	7.7	102.9	7.7	90.1	4.7	0.019	0.033	0.9982	50.9
Eu	105.1	3.3	102.6	7.2	92.4	3.7	0.007	0.021	0.9960^c^	24.3
EuN	107.5	6.1	102.6	5.6	90.7	3.6	0.008	0.025	0.9988	35.6
Hs	122.1	4.5	106.3	3.4	94.4	1.6	0.010	0.026	0.9978	39.2
HsN	97.9	7.3	104.4	3.4	96.6	3.1	0.008	0.023	0.9946	40.0
Ht	102.9	2.8	101.4	5.8	90.4	3.5	0.007	0.021	0.9980^d^	35.3
HtN	107.5	2.2	100.4	4.5	91.6	3.2	0.009	0.012	0.9986	25.9
Ic/Im	104.8	5.9	102.0	7.0	95.7	2.5	0.007	0.019	0.9980	23.3
IcN/LyN	105.9	6.0	97.2	5.7	88.2	1.5	0.015	0.030	0.9994^c^	27.2
Ig	97.1	6.8	94.4	2.8	94.6	3.9	0.012	0.030	0.9960	61.5
IgN	113.4	6.1	104.9	6.5	96.3	4.3	0.022	0.027	0.9980	33.1
ImN	114.1	7.7	102.0	5.9	92.6	3.5	0.006	0.015	0.9996	33.8
Jb	107.3	4.0	127.0	5.7	114.2	2.5	0.017	0.039	0.9984^c^	58.3
JbN	107.8	3.7	98.1	3.4	90.0	1.6	0.007	0.019	0.9990	39.1
Jl	-^e^	-^e^	97.1	5.9	91.0	2.4	0.023	0.056	0.9984	40.4
JlN	111.4	6.0	97.1	5.0	87.2	3.1	0.011	0.022	0.9982	43.3
Jn	65.1	7.4	67.7	9.1	64.4	2.0	0.023	0.036	0.9962	44.4
Lc	92.9	9.8	108.6	12.3	79.0	1.3	0.015	0.043	0.9940	74.0
LcN	108.3	5.2	108.4	5.8	94.9	2.7	0.010	0.021	0.9988	25.5
Ly	111.7	6.4	105.8	7.3	97.0	3.7	0.010	0.016	0.9980	25.6
Mc	96.8	4.3	97.4	7.1	92.7	1.4	0.013	0.040	0.9986	34.3
McN	98.6	1.6	92.3	5.3	84.3	4.1	0.010	0.016	0.9958	48.0
Mk	69.0	13.1	79.2	7.9	74.8	2.6	0.020	0.044	0.9992	41.8
MkN	-^e^	-^e^	12.5	27.3	13.4	14.6	0.013	0.101	0.9986	29.3
Mx	-^e^	-^e^	121.0	4.6	103.4	3.2	0.039	0.123	0.9974	57.7
MxN	175.6	5.8	172.2	3.6	156.9	6.3	0.012	0.037	0.9952	41.5
Rl	92.1	13.4	81.3	5.5	76.4	2.4	0.011	0.038	0.9982	56.0
RlN	35.5	8.8	32.5	6.5	36.2	2.8	0.010	0.025	0.9986	24.3
Rr	103.3	2.0	98.9	3.8	91.7	3.2	0.005	0.014	0.9990^c^	43.8
Rs	100.5	6.8	99.4	6.3	90.8	2.0	0.012	0.039	0.9976	48.9
RsN	103.1	9.9	99.6	7.8	90.3	1.7	0.013	0.016	0.9992	34.5
Sc	103.6	3.6	96.3	4.5	89.5	2.4	0.008	0.015	0.9934	52.9
ScN	116.9	12.5	99.8	7.1	92.8	7.2	0.017	0.039	0.9984^c^	32.4
Sco	105.3	6.1	102.1	4.2	92.7	3.7	0.009	0.027	0.9980	41.3
Sd	92.6	12.2	88.8	6.6	80.3	2.9	0.040	0.040	0.9990	44.2
Sk	93.0	9.8	99.4	3.2	93.5	2.9	0.011	0.034	0.9998^c^	41.7
Sl	-^e^	-^e^	96.8	5.3	91.1	2.2	0.054	0.054	0.9986	39.5
SlN	114.3	1.9	101.1	3.7	92.6	2.6	0.047	0.047	0.9986	38.8
Sp	103.1	3.2	95.2	5.5	84.8	3.4	0.011	0.032	0.9968	49.0
SpN	68.4	7.9	65.1	8.2	65.5	4.2	0.032	0.032	0.9988	27.8
Sv	103.3	6.9	98.2	3.0	91.6	3.1	0.009	0.019	0.9960	57.2
SvN	95.2	12.3	102.0	6.0	95.4	4.0	0.022	0.022	0.9988	44.0
Td	103.4	8.8	100.6	5.4	92.2	1.9	0.041	0.041	0.9972	43.9
Us	104.3	9.4	100.9	7.7	91.1	3.3	0.011	0.034	0.9970	42.1
UsN	99.3	6.9	97.7	7.0	86.4	3.8	0.009	0.028	0.9992^c^	46.6

^a^For the abbreviations of the analytes, see Table 1

^b^For calculation, see Eq. 3

^c^Linear up to 3.7 µg/L according to Mandel’s *F*-test

^d^Linear up to 2.8 µg/L according to Mandel’s *F*-test

^e^Concentration level below LOQ

**Fig. 3:**
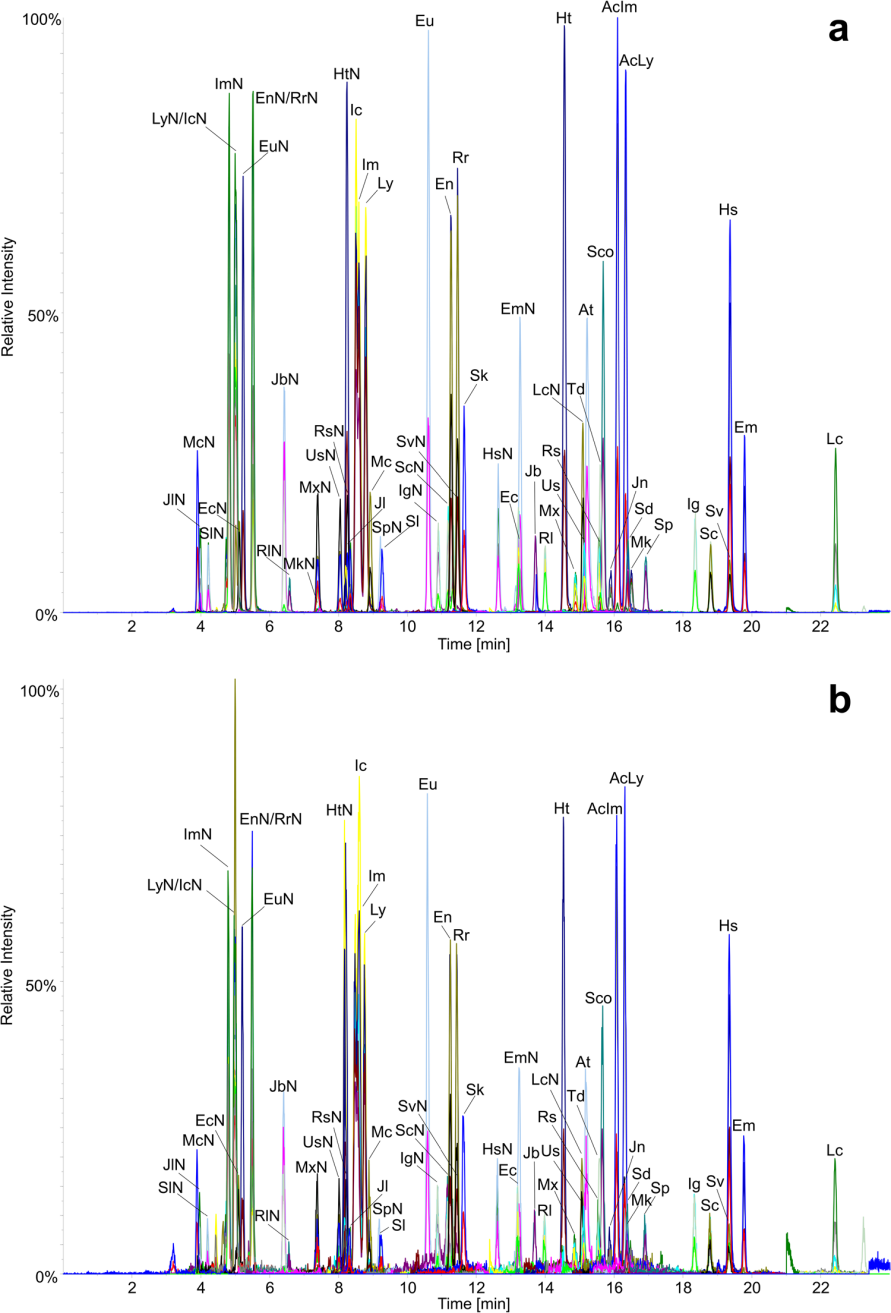
LC–MS/MS chromatograms of milk samples artificially contaminated with 30 pyrrolizidine alkaloids, 24 pyrrolizidine alkaloid *N*-oxides, and two tropane alkaloids to concentrations of 0.50 µg/L (**a**) and 0.05 µg/L (**b**) per individual analyte, prepared and measured using the final method. For abbreviations of the analytes, see Table 1

Chromatograms of milk samples spiked to individual PA/PANO/TA concentrations of 0.50 µg/L and 0.05 µg/L are presented in Fig. 3.

The recovery rates of 47 of the 56 examined analytes ranged between 76.4 and 116.9% at all tested concentration levels (Table 3). Furthermore, six analytes (seneciphylline-*N*-oxide, jaconine, merenskine, heliosupine, jacobine, and merepoxine) had recoveries within 65.1 to 127.0% at all tested levels and therefore slightly under or above the required 70 to 120% recovery rate mentioned in SANTE/12682/2019 (Table 3). For merepoxine-*N*-oxide, continuously high recovery rates (156.9 to 175.6%) were received. In contrast, riddelliine-*N*-oxide and merenskine-*N*-oxide showed poor recovery rates of 32.5 to 36.2% and 12.5 to 13.4%, respectively. Consequently, the determination of merenskine-*N*-oxide, merepoxine-*N*-oxide, and riddelliine-*N*-oxide was considered to be semiquantitative. In case of jacoline, merenskine-*N*-oxide, merepoxine, and sceleratine, the LOQ was higher than 0.05 µg/L, and therefore no recovery or repeatability rates were assessed for these analytes at the lowest spike level.

Overall, PA/PANO with a chlorine atom, namely jaconine, merenskine, and merenskine-*N*-oxide, showed a lower recovery, whereas their corresponding PA/PANO with an epoxide group, i.e., jacobine, merepoxine, and merepoxine-*N*-oxide, showed slightly or even notably (merepoxine-*N*-oxide) increased recovery rates (Table 3). This might be due to a transformation of the chlorine-incorporating PA/PANO to the respective epoxide analytes under alkaline conditions during elution and evaporation. This reaction is already known for the second step of the so-called chlorohydrin method in epoxide production [50]. To prove this hypothesis, further investigations are still needed.

In general, riddelliine-*N*-oxide and seneciphylline-*N*-oxide showed lower recoveries compared to other compounds included in the method (Table 3). This is analogous to the recovery data published by Mulder et al. for riddelliine-*N*-oxide (45%) and seneciphylline-*N*-oxide (74%), herein being the analytes with the lowest recoveries [21]. As there were C18 cartridges used, the lower recovery of these two PANO presumably was likely due to the extraction procedure. When examining the impact of the n-hexane addition during the extraction step, no differences in the recovery or response of riddelliine-*N*-oxide and seneciphylline-*N*-oxide (ESM Table S3) were observed. Thus, n-hexane extraction seemed not to cause the observed decreased recoveries of some analytes.

The TA atropine and scopolamine showed good recovery rates (87.0 to 105.3%), comparable to those achieved for most of the PA/PANO (76.4 to 116.9%) (Table 3).

Repeatability expressed as the RSD_r_ was good, showing values below 10% at spiking levels of 0.05 µg/L and 0.50 µg/L for 49 of the 56 analytes. Namely merenskine, riddelliine, spartioidine, sceleratine, senecionine-*N*-oxide, and senecivernine-*N*-oxide had RSD_r_ values between 10 and 15% at the lowest spike level (0.05 µg/L). For lasiocarpine and merenskine-*N*-oxide, the calculated RSD_r_ at the 0.5 µg/L spike level was 12.3% and 27.3%, respectively (Table 3). At the highest artificial contamination level (3.00 µg/L), the repeatability rates were even below 5% for 53 of the 56 analytes and below 15% for senecionine-*N*-oxide, merenskine-*N*-oxide, and merepoxine-*N*-oxide. In total, the method’s repeatability was in accordance to SANTE/12682/2019 for all analytes except merenskine-*N*-oxide at the 0.50 µg/L level. Hence, the six PA/PANO (seneciphylline-*N*-oxide, jaconine, merenskine, heliosupine, jacobine, and merepoxine) showed recovery rates in the range of 65.1 to 127.0%. Although these values lay slightly outside the 70 to 120% recovery range required by SANTE/12682/2019, the six PA/PANO were also regarded to be quantitatively assessable due to their good RSD_r_ values, ranging from 1.6 to 13.1% (Table 3).

LOD and LOQ of the method were assessed according to German DIN 32,645 and ranged from 0.005 to 0.054 µg/L and 0.009 to 0.123 µg/L (Table 3), respectively. The low LOQ values were additionally confirmed with the recovery and repeatability values obtained from the lowest tested level of 0.05 µg/L. Therefore, the developed method was very sensitive and fulfilled the requirements for an analysis method for the determination of PA/PANO and TA in milk. This is highly important as these toxic contaminants are mostly present in low amounts in milk [21]. The LOQ values of the developed method were in the same concentration range of the previously published methods with good sensitivity (detailed information in ESM Table S1). For comparison, Mulder et al. reported a LOQ of 0.1 µg/L, Huybrechts and Callebaut LOQ values from 0.003 to 0.033 µg/kg, and Chung and Lam LOQ values from 0.010 to 0.087 µg/kg for PA/PANO [21, 24, 44]. Lamp et al. reported a LOQ of 0.075 µg/kg for a method developed only for atropine and scopolamine [20].

Linearity was given up to a concentration of 5.5 µg/L for 43 of the 56 analytes. Further twelve analytes were categorised as linear up to a concentration of 3.7 µg/L. Detection of heliotrine, one of the analytes for which the method was most sensitive, was linear up to a concentration of 2.8 µg/L according to Mandel’s *F*-test. The corresponding *R*^2^ values were > 0.993 for all analytes (Table 3). Additionally, the back-calculated value of the bracketing calibration was within ± 20% of the assigned concentration and therefore was in accordance to the criteria of SANTE/12682/2019. The matrix effects manifested in decreased signal intensities. Matrix suppression ranged between 23.3% for intermedine/indicine and 74.0% for the latest eluting compound, lasiocarpine. This indicated the need for matrix-matched calibration, particularly for the later eluting PA/PANO compounds, to correct for these matrix effects during LC–MS/MS measurement. Isotopically labelled internal standards can be used to correct for losses during extraction and clean-up procedures and specific matrix interference, but these standards are expensive and not commercially available for most of the PA/PANO.

Overall, 51 of the 54 examined PA/PANO as well as the TA atropine and scopolamine were successfully validated according to SANTE/12682/2019. Solely the results for riddelliine-*N*-oxide, merenskine-*N*-oxide, and merepoxine-*N*-oxide indicated only semiqualitative analysis of these compounds. To the best of our knowledge, merenskine-*N*-oxide and merepoxine-*N*-oxide were not included in any previously published method for the determination of PA/PANO in milk, although they were supposed to naturally occur in *Senecio* species [51].

### Method’s applicability

Ten raw and five pasteurised milk samples were analysed to prove the method’s applicability. PA were detected in three milk samples, comprising both raw and pasteurised milk (Table 4).

**Table 4:** Occurrence of pyrrolizidine alkaloids in raw milk from vending stations (*n* = 10) and pasteurised milk from regional marketers (*n* = 5) in Bavaria

Sample	Erucifoline [µg/L]	Lycopsamine [µg/L]	Retrorsine [µg/L]	Senkirkine [µg/L]	Sum^a^ [µg/L]
Raw milk 1	n.d	n.d	n.d	n.d	n.d
Raw milk 2	n.d	n.d	< 0.039	n.d	0.020
Raw milk 3	n.d	n.d	n.d	n.d	n.d
Raw milk 4	n.d	n.d	n.d	n.d	n.d
Raw milk 5	n.d	n.d	n.d	n.d	n.d
Raw milk 6	n.d	n.d	n.d	n.d	n.d
Raw milk 7	n.d	n.d	n.d	n.d	n.d
Raw milk 8	n.d	n.d	n.d	n.d	n.d
Raw milk 9	n.d	< 0.016	n.d	n.d	0.008
Raw milk 10	n.d	n.d	n.d	n.d	n.d
Pasteurised milk 1	n.d	n.d	n.d	n.d	n.d
Pasteurised milk 2	n.d	n.d	n.d	n.d	n.d
Pasteurised milk 3	n.d	n.d	n.d	n.d	n.d
Pasteurised milk 4	n.d	n.d	n.d	n.d	n.d
Pasteurised milk 5	0.010	< 0.016	n.d	< 0.034	0.035

*n.d.*, not detected

^a^Pyrrolizidine alkaloids detected below the limit of quantification (LOQ) were considered with 0.5 times the LOQ value

In two of the ten raw milk samples, one PA was detected in minor amount above the LOD (Table 4 and for chromatograms ESM Fig. S4 and Fig. S5). Retrorsine was identified in raw milk 2 and lycopsamine was detected in raw milk 9. In one of the five pasteurised milks, three PA were detected (chromatograms in ESM Fig. S6 to Fig. S8). Erucifoline was detected at a concentration of 0.010 µg/L, and therefore in the range of its LOQ. Senkirkine and lycopsamine were also detected in this sample in trace amounts between their respective LOD and LOQ.

For additional confirmation of the results, blank milk was spiked to the calculated concentrations. The occurrence of lycopsamine, erucifoline, and senkirkine was confirmed in all replicates and in case of retrorsine in two out of three samples. Detailed information is presented in ESM Table S4.

The combined occurrence of erucifoline and senkirkine in pasteurised milk 5 indicated plants of the genus *Senecio* or *Jacobaea*, respectively, as the causative source for the feed contamination [19, 52]. This milk, labelled as hay milk, was obtained from a regional marketer in the Bavarian district Oberallgäu, an area known for its spread of marsh ragwort (*Jacobaea aquatica*). While lycopsamine is known to occur in plants of the families Boraginaceae, Apocynaceae, and in the tribus *Eupatorieae* (Asteraceae), retrorsine indicated the contamination of the feed with plants from the family Asteraceae (especially genus *Senecio* or *Jacobaea*) [4, 53]. Nevertheless, distinct determination of the exact botanical origin of contaminating plants based on PA/PANO pattern in milk is barely possible as it is already known that PA/PANO patterns shift greatly from plant material to milk due to individual carry-over rates of PA/PANO [18]. So far, typical PA/PANO patterns in milk were only described for supplementation with certain ragwort plants (*Jacobaea vulgaris* and *Senecio inaequidens*), common groundsel (*Senecio vulgaris*), and viper’s bugloss (*Echium vulgare*) [17–19].

In a previously conducted study, the occurrence of 19 PA and 16 PANO in 182 retail milk samples from several European countries was investigated [21]. In eleven of these samples, PA, namely senkirkine, otosenine, lycopsamine, echimidine, retrorsine, and jacoline, were identified with individual analyte concentrations ranging from 0.05 to 0.16 µg/L. Moreover, Huybrecht and Callebaut detected one PA/PANO each in eight out of 63 milk samples, namely lycopsamine, retrorsine-*N*-oxide, heliotrine, and senkirkine, up to a concentration of 0.061 µg/kg [24]. In contrast, Chung and Lam and Yoon et al. did not identify PA/PANO in any of the investigated goat milk and cow milk samples from Asia [23, 46]. In case of the method used by Yoon et al., LOD values ranged between 0.2 and 1.99 µg/L and therefore above all previously detected PA/PANO amounts in milk. Regarding TA, in a study conducted by Zheng et al. on the occurrence of two TA and two quinolizidine alkaloids in ten milk samples, LOD values for TA were also in the range of 0.4 to 1 µg/kg and no TA were detected [25].

Until now, PA/PANO analysis in milk and milk products was focused on retail products obtained from supermarkets [21, 23, 24]. However, the consumers’ interest in locally produced food has increased in the last years [54]. Regarding milk, this consumer demand is met, e.g., by direct sales through milk vending machines at dairy farms. In case of industrially processed milk, PA/PANO amounts contained in an individual cow’s milk are expected to get highly diluted due to milk derived from several dairy farms and large processed volumes. In contrast, this does not apply if milk is sold directly from a dairy farm. Hence, higher contamination levels in milk from direct marketers than in retail milk might be possible. In this small study, we found PA in three out of 15 milk samples up to a sum content of 0.035 µg/L and thus being comparable to those reported for retail milk [21]. Assuming a PA/PANO concentration of 0.04 µg/L, for a toddler (2 to < 5 years, 16.15 kg) with an average daily milk intake of 230.4 g/day, this would result in a PA/PANO exposure of 0.64 ng/kg body weight (bw)/day via milk. Based on a BMDL_10_ of 237 µg/kg bw/day, this corresponds to a margin of exposure (MOE) of > 400,000; thus, it is unlikely to pose a health concern [3, 55].

Up to now, the occurrence of TA in milk was a minor issue in research. On the basis of data available in 2013, the European Food Safety Authority (EFSA) concluded that TA exposure via milk consumption was not of concern for human health [12, 56]. A recently published study has demonstrated the transfer of atropine and scopolamine to milk in minor amounts, comparable to the transfer rates of PA/PANO [20]. On this basis, we included the two most common TA atropine and scopolamine into the scope of our method. Herewith, data on the occurrence of atropine and scopolamine in milk can be acquired simultaneously with the quantitative determination of PA/PANO with minimal additional effort.

## Conclusion

A sensitive LC–MS/MS method for the quantitative determination of 51 PA/PANO and the two TA atropine and scopolamine was developed and validated for raw cow’s milk. Furthermore, three additional PANO were included for semiquantitative determination. The HPLC separation under alkaline conditions enabled us to distinguish nearly all isomeric compounds within a total run time of 31 min including a 7-min re-equilibration time for the column. The extraction and clean-up procedure based on PCX cartridges showed very good recovery and precision for most of the included analytes without the necessity of time-consuming freeze-out or pH adjustments steps. Validation of the method confirmed very low LOD and LOQ values which are crucial for PA/PANO and TA analysis in milk. Assessed matrix effects still indicated the need of matrix-matched calibration for reliable quantification. Covering 54 PA/PANO, the method allowed determination of the PA/PANO composition which may provide valuable information on the botanical origin of the contamination in the feed chain. The incorporation of atropine and scopolamine allowed to simultaneously screen for TA in raw milk and to generate first data on their potential occurrence in such samples with minimal additional effort. The investigation of ten raw milk and five pasteurised milk samples demonstrated the excellent applicability on real samples. It also revealed the presence of PA in three of the 15 samples, while neither PANO nor TA were detected. The determined results were close to the LOD and LOQ. The PA profile in the contaminated milk samples indicated plant material of the plant families Boraginaceae and Asteraceae (genus *Senecio* or *Jacobaea*) to have caused the detected contaminations.

## Supplementary Information

Below is the link to the electronic supplementary material.

Supplementary file1 (PDF 906 KB)

## Abbreviations

AcIm: Acetylintermedine
AcImN: Acetylintermedine-*N-*oxide
AcLy: Acetyllycopsamine
AcLyN: Acetyllycopsamine-*N-*oxide
At: Atropine
BMDL_10_: Benchmark dose lower confidence limit 10%
BW: Bodyweight
Ec: Erucifoline
EcN: Erucifoline-*N-*oxide
Em: Echimidine
EmN: Echimidine-*N-*oxide
En: Echinatine
EnN: Echinatine-*N-*oxide
Eu: Europine
EuN: Europine-*N-*oxide
EFSA: European Food Safety Authority
ESI: Electrospray ionisation
ESI +: Positive electrospray ionisation
HILIC: Hydrophilic interaction liquid chromatography
HSOS: Hepatic sinusoidal obstruction syndrome
HPLC: High-performance liquid chromatography
Hs: Heliosupine
HsN: Heliosupine-*N-*oxide
Ht: Heliotrine
HtN: Heliotrine-*N-*oxide
Ic: Indicine
IcN: Indicine-*N-*oxide
Ig: Integerrimine
IgN: Integerrimine-*N-*oxide
Im: Intermedine
ImN: Intermedine-*N-*oxide
Jb: Jacobine
JbN: Jacobine-*N-*oxide
Jl: Jacoline
JlN: Jacoline-*N-*oxide
Jn: Jaconine
Lc: Lasiocarpine
LcN: Lasiocarpine-*N-*oxide
LC–MS/MS: Liquid chromatography tandem mass spectrometry
LLE: Liquid-liquid extraction
LOD: Limit of detection
LOQ: Limit of quantification
Ly: Lycopsamine
LyN: Lycopsamine-*N-*oxide
Mc: Monocrotaline
McN: Monocrotaline-*N-*oxide
Mk: Merenskine
MkN: Merenskine-*N-*oxide
MOE: Margin of exposure
MRM: Multiple reaction monitoring
Mx: Merepoxine
MxN: Merepoxine-*N-*oxide
PA: Pyrrolizidine alkaloid
PAH: Pulmonary arterial hypertension
PANO: Pyrrolizidine alkaloid *N-*oxide
PCX: Polymeric cation exchange
PFP: Pentafluorophenyl
PVDF: Polyvinylidene difluoride
Rl: Riddelliine
RlN: Riddelliine-*N-*oxide
Rr: Rinderine
RrN: Rinderine-*N*-oxide
RP: Reversed phase
Rs: Retrorsine
RsN: Retrorsine-*N-*oxide
RSD: Relative standard deviation
RSD_r_: Repeatability
S/N: Signal-to-noise ratio
Sc: Senecionine
ScN: Senecionine-*N-*oxide
Sco: Scopolamine
Sd: Spartioidine
Sl: Sceleratine
SlN: Sceleratine-*N-*oxide
Sp: Seneciphylline
SPE: Solid-phase extraction
SpN: Seneciphylline-*N-*oxide
Sk: Senkirkine
Sv: Senecivernine
SvN: Senecivernine-*N-*oxide
TA: Tropane alkaloid
Td: Trichodesmine
UHPLC: Ultra high-performance liquid chromatography
Us: Usaramine
UsN: Usaramine-*N*-oxide
VOD: Veno-occlusive disease
