# Correction to: A sensitive LC–MS/MS method for isomer separation and quantitative determination of 51 pyrrolizidine alkaloids and two tropane alkaloids in cow’s milk

**DOI:** 10.1007/s00216-022-04454-0

**Published:** 2022-11-22

**Authors:** Lisa Monika Klein, Angelika Miriam Gabler, Michael Rychlik, Christoph Gottschalk, Florian Kaltner

**Affiliations:** 1 Chair of Food Safety and Analytics, Faculty of Veterinary Medicine, Ludwig Maximilian University of Munich, Schoenleutnerstr. 8, 85764 Oberschleissheim, Germany; 2 Chair of Analytical Food Chemistry, TUM School of Life Sciences Weihenstephan, Technical University of Munich, Maximus‑von‑Imhof‑Forum 2, 85354 Freising, Germany; 3 Present Address: Unit Plant Toxins and Mycotoxins, Department Safety in the Food Chain, German Federal Institute for Risk Assessment, Max‑Dohrn‑Str. 8‑10, 10589 Berlin, Germany; 4 Present Address: Institute of Food Chemistry and Food Biotechnology, Justus Liebig University of Giessen, Heinrich‑Buff‑Ring 17‑19, 35392 Giessen, Germany

**Correction to: Analytical and Bioanalytical Chemistry (2022) 414:8107–8124**

https://doi.org/10.1007/s00216-022-04344-5

The original article contained a mistake.

The authors regret to inform that an error has been detected in the text of the results and discussion section “Final method for the determination of PA/PANO and TA in milk”. Instead of “After washing with 10 mL 2% aqueous formic acid and 10 mL of methanol, the analytes were eluted with 6 mL of ammoniated methanol (5%) into a glass vial” it should have been “After washing with 10 mL water and 10 mL of methanol, the analytes were eluted with 6 mL of ammoniated methanol (5%) into a glass vial”.

