# Supplementary material for: A sensitive LC–MS/MS method for isomer separation and quantitative determination of 51 pyrrolizidine alkaloids and two tropane alkaloids in cow’s milk
Source: Anal Bioanal Chem. 2022 Oct 1;414(28):8107–24. doi: 10.1007/s00216-022-04344-5 (PMC9613554; doi:10.1007/s00216-022-04344-5)
Supplement: Supplementary file 1 — Supplementary file1 (PDF 906 KB) [file 216_2022_4344_MOESM1_ESM.pdf]

A sensitive LC-ESI-MS/MS method for isomer separation and quantitative determination of 51 pyrrolizidine alkaloids and two tropane alkaloids in cow's milk

Electronic Supplementary Material (ESM)

Lisa Monika Klein[1,2] *, Angelika Miriam Gabler[1], Michael Rychlik[2], Christoph Gottschalk[1,♦], Florian Kaltner[1,►]

[1] Chair of Food Safety and Analytics, Faculty of Veterinary Medicine, Ludwig Maximilian University of Munich, Schoenleutnerstr. 8, 85764 Oberschleissheim, Germany

[2] Chair of Analytical Food Chemistry, TUM School of Life Science Weihenstephan, Technical University of Munich, Maximus-von-Imhof-Forum 2, 85354 Freising, Germany

[♦] Present address: Unit Plant toxins and Mycotoxins, Department Safety in the Food Chain, German Federal Institute for Risk Assessment, Max-Dohrn-Str. 8-10, 10589 Berlin, Germany

[►] Present address: Institute of Food Chemistry and Food Biotechnology, Justus Liebig University of Giessen, Heinrich-Buff-Ring 17-19, 35392 Giessen, Germany

* Corresponding author

E-mail: lisa.klein@ls.vetmed.uni-muenchen.de

Table S1 Overview of previously published extraction and clean-up procedures, and (LC-)MS/MS methods applied for the determination of pyrrolizidine alkaloids (PA), pyrrolizidine alkaloid *N*-oxides (PANO) and tropane alkaloids (TA) in milk and milk products

Analytes	Sample volume [mL or g]	Extraction and clean-up	Mobile phase	Stationary phase	LC-MS/MS instrumentation	Recovery [%]	LOQ [µg/kg or µg/L]	Ref.
22 PA<br>14 PANO	3	1) Freeze-out (-20 °C, >4 h)<br>2) LLE (methanol with 0.1% formic acid)<br>3) Freeze-out (-20 °C, >4 h)<br>4) Centrifugation (1,950 ×*g*, 4 °C, 10 min)<br>6) Evaporation, reconstitution (H_2O with 0.1% acetic acid)<br>7) Centrifugation (1950 ×*g,* 10 min) and filtration (0.45 µm)	A: H_2O with 0.1% acetic acid<br>B: Acetonitrile	Acqutiy BEH C18 (100 x 2.1 mm, 1.7 µm)	UHPLC-QqQ-MS/MS	n/a	0.05 - 0.2	[1] According to in-house validated protocol (van den Top H. and Schothorst R., 2007)
10 PA<br>4 PANO	4	1) LLE (0.05 mol/L H_2SO_4, vortex mixing, 30 s)<br>2) Centrifugation (3,421 ×*g*, 10 min)<br>3) Volume adjustment in flask<br>4) SPE (SCX, Strata X-C)<br>5) Evaporation, reconstitution (methanol) and syringe filtration	A: H_2O with 0.05% formic acid and acetonitrile (95/5, v/v)<br>B: Acetonitrile	Kinetex PFP (150 x 2.1 mm, 2.6 µm)	(U)HPLC-QqQ-MS/MS	65 - 125	3.5	[2]
10 PA<br>4 PANO	10	1) LLE (acetonitrile with 1% acetic acid, vortex mixing, 30 s)<br>2) Addition of sodium acetate, anhydrous magnesium sulphate and acetonitrile with 1% acetic acid and shaken (1 min)<br>3) Centrifugation (3,421 ×*g*, 10 min)<br>4) Addition of primary and secondary exchange material, carbon and anhydrous magnesium sulphate (dSPE, vortex mixing, 1 min)<br>5) Centrifugation (3,421 ×*g*, 10 min)<br>6) (a) Evaporation, reconstitution (methanol) and syringe filtration (0.22 µm, PTFE)<br>(b) Only syringe filtration (0.22 µm, PTFE)	A: H_2O with 0.05% formic acid and acetonitrile (95/5, v/v)<br>B: Acetonitrile	Kinetex PFP (150 x 2.1 mm, 2.6 µm)	(U)HPLC-QqQ-MS/MS	(a) 17 - 73,<br>(b) 18 - 74	3.5	[2]
9 PA	10	1) LLE (chloroform-methanol, 1:1, v/v, sonication, 40 min)<br>2) Filtration, evaporation and reconstitution (methanol)<br>3) Freeze-out (-24 °C, 30 min) and filtration<br>4) Reconstitution (methanol) of the precipitated lipids<br>5) Freeze-out (-24 °C, 30 min) and filtration<br>6) Concentration of the extract (rotary evaporation)<br>7) SPE (SCX, Strata X-C)<br>8) Evaporation, reconstitution (methanol)	A: H_2O with 0.1% formic acid<br>B: Acetonitrile	HECTOR-M C18 (150 x 4.6 mm, 3.0 µm)	HPLC-QqQ-MS/MS (ESI+)	83 - 100	0.23 - 1.76	[3]

Table S1 (*continued*)

Analytes	Sample volume [mL or g])	Extraction and clean-up	Mobile phase	Stationary phase	LC-MS/MS instrumentation	Recovery [%]	LOQ [µg/kg or µg/L]	Ref.
10 PA<br>6 PANO	20	1) LLE (37% HCl and hexane, 60 °C, 60 min)<br>2) Centrifugation (13,000 ×*g*, 5 min) and syringe filtration	A: H_2O with 5 mmol/L ammonium acetate and 0.05% acetic acid<br>B: Acetonitrile	Kinetex XB-C18 (100 x 4.6 mm, 2.6 µm)	UHPLC-QqQ-MS/MS (ESI+)	89 - 114	0.003 - 0.033	[4]
19 PA<br>16 PANO	3	1) LLE (0.2% formic acid and hexane, shaking, 30 min)<br>2) Centrifugation (3,500 *g*, 15 min)<br>3) Basification (ammonia (25%), pH 9 - 10)<br>4) Centrifugation (3,500 ×*g*, 15 min)<br>5) SPE (C18, Strata X)<br>6) Evaporation, reconstitution (methanol/H_2O, 10/90, v/v) and filtration (0.45 µm, PTFE)	A: H_2O with 6.5 mmol/L ammonium hydroxide<br>B: Acetonitrile with 1.2 mmol/L ammonium hydroxide	Acqutiy BEH C18 (150 x 2.1 mm, 1.7 µm)	UHPLC-Qtrap-MS/MS (ESI+)	45 - 107	0.1	[5], Adapted by [6], [7]
15 PA<br>13 PANO	2	1) LLE (0.05 mol/L H_2SO_4, sonication, 15 min)<br>2) Centrifugation (8,500 rpm, RT, 10 min)<br>3) Neutralisation (aqueous ammonia, pH 6 - 7)<br>4) Freeze-out (-80 °C, >18 h)<br>5) Centrifugation (8,500 rpm, 10 °C, 10 min)<br>6) Syringe filtration (0.45 µm, RC)<br>7) SPE (C18, Supleco DSC-C18, 2-step elution)<br>6) Evaporation, reconstitution (H2O with 0.5 mmol/L ammounium formate and 2 mmol/L formic acid) and syringe filtration (0.2 µm, RC)	A: Acetonitrle/H_2O (80/20, v/v) with 0.5 mmol/L ammonium formate and 2 mmol/L formic acid<br>B: H_2O with 0.5 mmol/L ammonium formate and 2 mmol/L formic acid	Aquity CSH C18 (150 x 2.1 mm, 1.7 µm)	UHPLC-Qtrap-MS/MS (ESI+)	57 - 120	0.010 - 0.087	[8]
6 PA	5	1) Dilution (0.5% formic acid)<br>2) Centrifugation (10,000 rpm, 10 min, twice)<br>3) pH adjustment to 10 - 11<br>4) LLE (dichloromethane, shaking, twice)<br>5) Evaporation of combined dichlormethane phase (N_2) and reconstitution (methanol/H_2O, 50/50, v/v)	/	/	DART-IT-MS (positive mode)	89.3 - 112.1	1.83 - 2.82	[9]

Table S1 (*continued*)

Analytes	Sample volume [mL or g])	Extraction and clean-up	Mobile phase	Stationary phase	LC-MS/MS instrumentation	Recovery [%]	LOQ [µg/kg or µg/L]	Ref.
2 TA (2 QA)	2	1) LLE (0.5 mol/L EDTA, acetonitrile with 0.5% triflouroacetic acid, vortex mixing)<br>2) Addition of magnesium sulphate, sodium chloride, sodium citrate tribasic dihydrate, sodium citrate tribasic sesquihydrate (vortex mixing, 5 min)<br>3) Centrifugation (2,600 ×*g*, RT, 10 min)<br>4) Addition of C18 sorbent material and magnesium sulfate to supernatant (dSPE, vortex mixing, 5 min)<br>5) Centrifugation (2,600 ×*g*, RT, 10 min)<br>6) Evaporation, reconstitution (methanol), centrifugation (10,840 *g)* and syringe filtration (0.2 µm)	A: H_2O with 0.1% formic acid and 10 mmol/L ammonium formate<br>B: methanol	Kinetex EVO C18	HPLC-QqQ-MS/MS (ESI+)	81 - 97	2 - 5	[10]
2 TA	2	1) LLE (methanol with 1% formic acid, vortex mixing, 1 min)<br>2) Centrifugation (4,200 ×*g*, 4 °C)<br>3) Freeze-out (-20 °C, >12 h)<br>4) Centrifugation (18,000 ×*g*, 10 min, -10 °C)	A: H_2O with 0.1% formic acid<br>B: Methanol	Luna Phenyl-Hexyl (150 x 2 mm, 5.0 µm)	HPLC-Qtrap-MS/MS (ESI+)	96 - 99	0.075	[11]

Abbreviations: DART direct analysis in real time, *dSPE* dispersive solid phase extraction, *ESI*+ electrospray ionization positive mode, *IT* ion trap, *LLE* liquid-liquid extraction, *LOQ* limit of quantification, *MS* mass spectrometry, *MS/MS* tandem mass spectrometry, *PFP* pentafluorophenyl, *PTFE* polytetrafluorethylen, *QA* quinolizidine alkaloids, *QqQ* triple quadrupole, *Qtrap* quadrupole-linear ion trap mass spectrometer, *RC* regenerated cellulose, *Ref.* references, *SCX* strong cation exchange, *SPE* solid phase extraction, *UHPLC* ultra high performance liquid chromatography

Table S2 Additional information on milk samples analysed for method development, validation and field sample testing

Sample Name	Labelling	Sample Type	Source of Supply	Bavarian Province of Origin	Production	Sampling Date [dd.mm.yy]	Expiration Date [dd.mm.yy]
Blank milk		Raw milk	Milk filling station	Upper Bavaria	Organic	16.06.20	-
Blank milk		Raw milk	Milk filling station	Upper Bavaria	Organic	07.10.20	-
Raw milk 1		Raw milk	Milk filling station	Upper Franconia	Conventional	31.03.21	-
Raw milk 2		Raw milk	Milk filling station	Upper Franconia	Conventional	03.04.21	-
Raw milk 3		Raw milk	Milk filling station	Upper Franconia	Organic	03.04.21	-
Raw milk 4		Raw milk	Milk filling station	Upper Franconia	Conventional	03.04.21	-
Raw milk 5		Raw milk	Milk filling station	Swabia	Conventional	29.04.21	-
Pasteurized milk 1	Hay milk	Pasteurised milk	Regional marketer	Upper Bavaria	Organic	10.05.21	03.05.21
Pasteurized milk 2	Hay milk	Pasteurised milk	Regional marketer	Upper Bavaria	Organic	10.05.21	01.05.21
Pasteurized milk 3	Fresh organic alpine milk	Pasteurised milk	Regional marketer	Upper Bavaria	Organic	10.05.21	04.05.21
Pasteurized milk 4	Fresh milk	Pasteurised milk	Regional marketer	Upper Bavaria	Organic	10.05.21	-
Pasteurized milk 5	Hay milk	Pasteurised milk	Regional marketer	Upper Bavaria	Conventional	20.07.21	24.07.21
Raw milk 6		Raw milk	Milk filling station	Upper Bavaria	Conventional	28.09.21	-
Raw milk 7		Raw milk	Milk filling station	Upper Bavaria	Conventional	28.09.21	-
Raw milk 8		Raw milk	Milk filling station	Upper Bavaria	Conventional	27.09.21	-
Raw milk 9		Raw milk	Milk filling station	Upper Bavaria	Conventional	02.10.21	-
Raw milk 10		Raw milk	Milk filling station	Upper Bavaria	Conventional	03.10.21	-

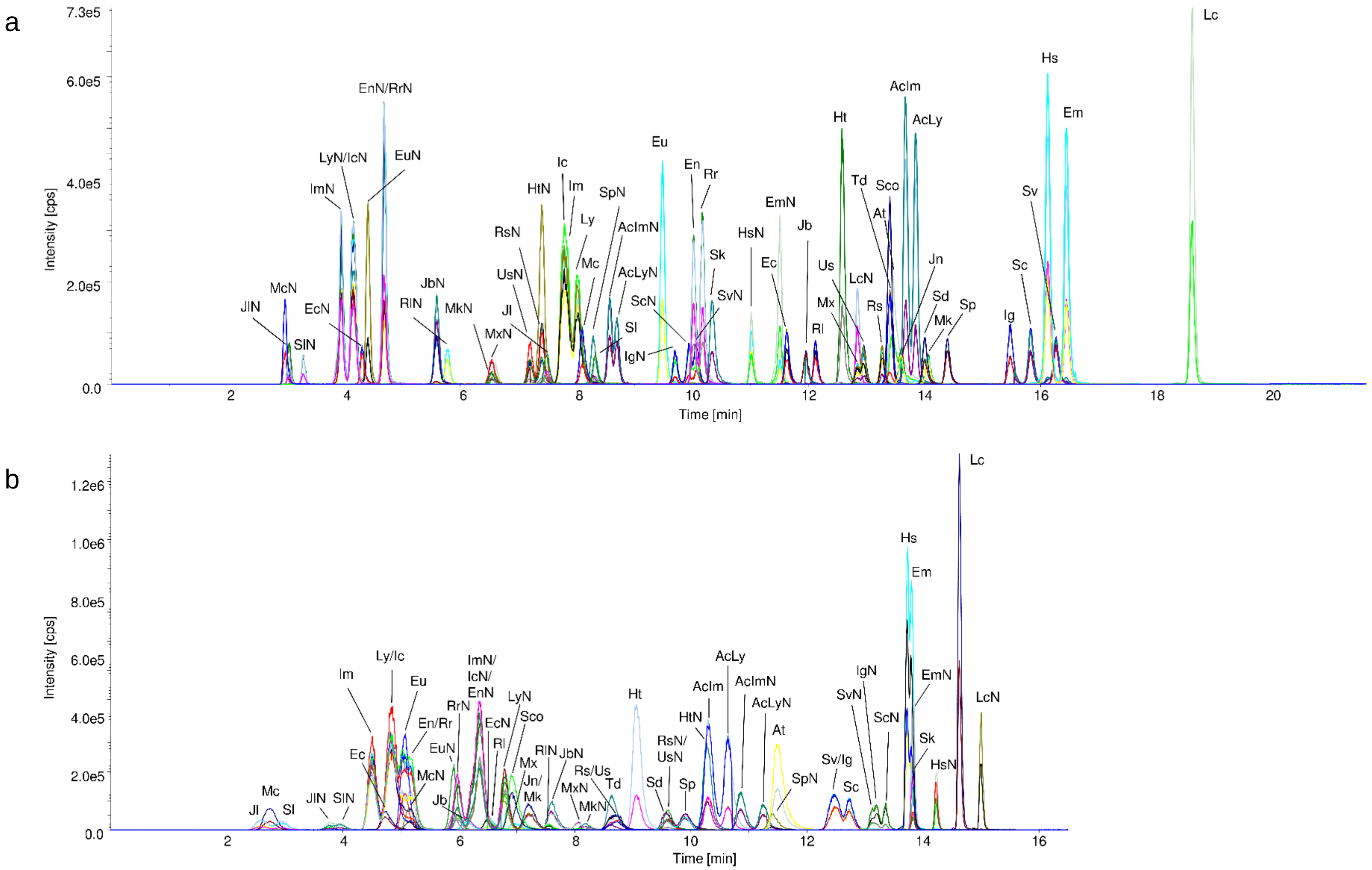

Fig. S1 Chromatographic separation of a 5 ng/mL standard solution of 56 pyrrolizidine alkaloids and two tropane alkaloids under alkaline (solvent A: 10 mmol/L ammonium carbonate in water, solvent B: acetonitrile, a) and acidic conditions (solvent A: water, solvent B: acetonitrile/water (95/5, v/v,), both containing 5 mmol/L ammonium formate and 26.5 mmol/L formic acid, b) solvent conditions using a 150 x 2.1 mm KinetexTM 5 µm EVO C18 column. For Abbreviations, see **Table 1**

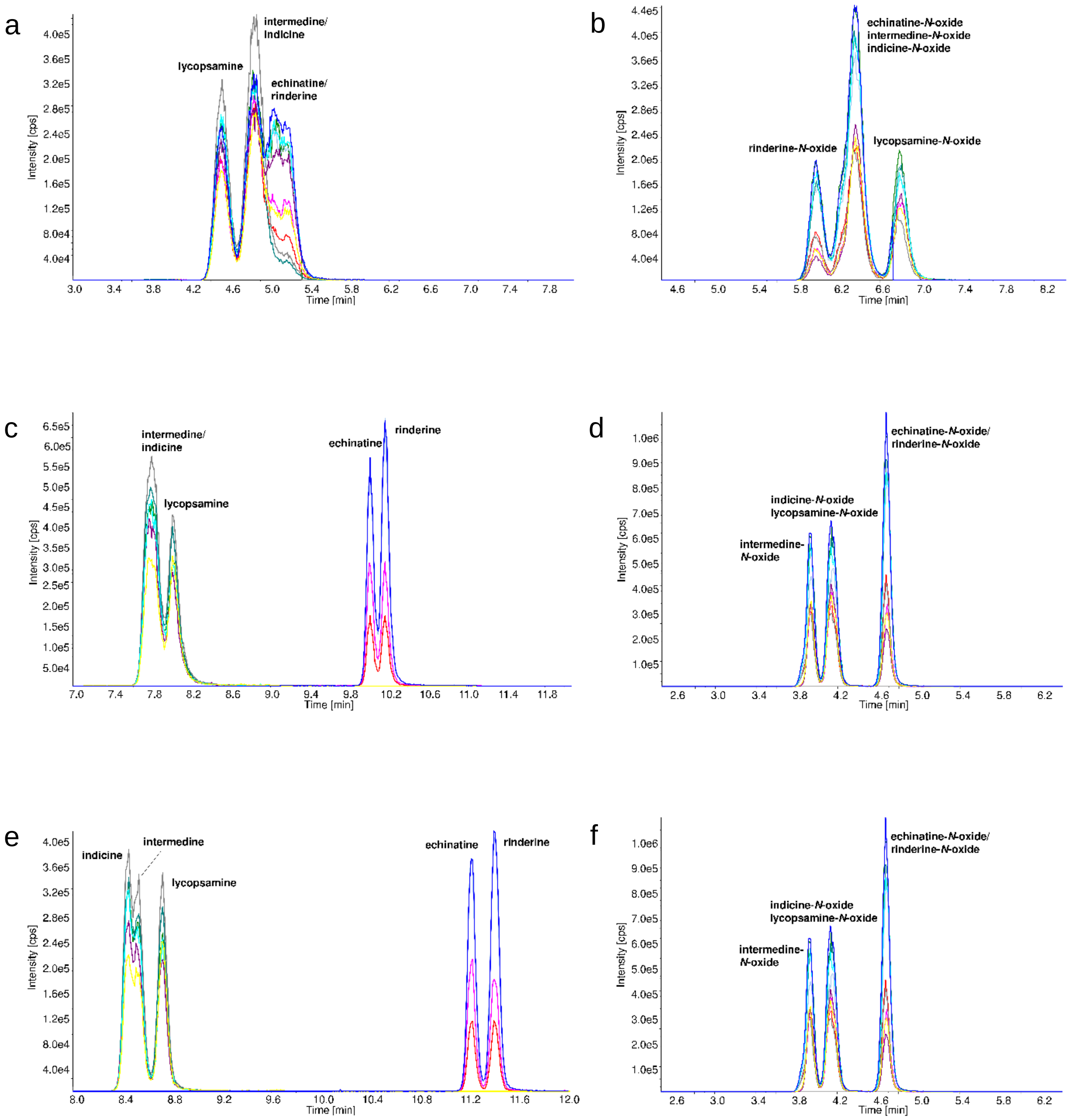

Fig. S2 Multiple reaction monitoring (MRM) chromatograms of isomeric pyrrolizidine alkaloids indicine, intermedine, lycopsamine, echniatine and rinderine and their corresponding *N*-oxides under acidic solvent conditions using a 150 x 2.1 mm KinetexTM 5 µm EVO C18 column (a + b) and alkaline solvent conditions using a 150 x 2.1 mm KinetexTM 5 µm EVO C18 column (c + d) and alkaline solvent conditions using a 100 x 2.1 mm KinetexTM 2.6 µm EVO C18 (e +f). Individual mass transitions are illustrated with different colours.

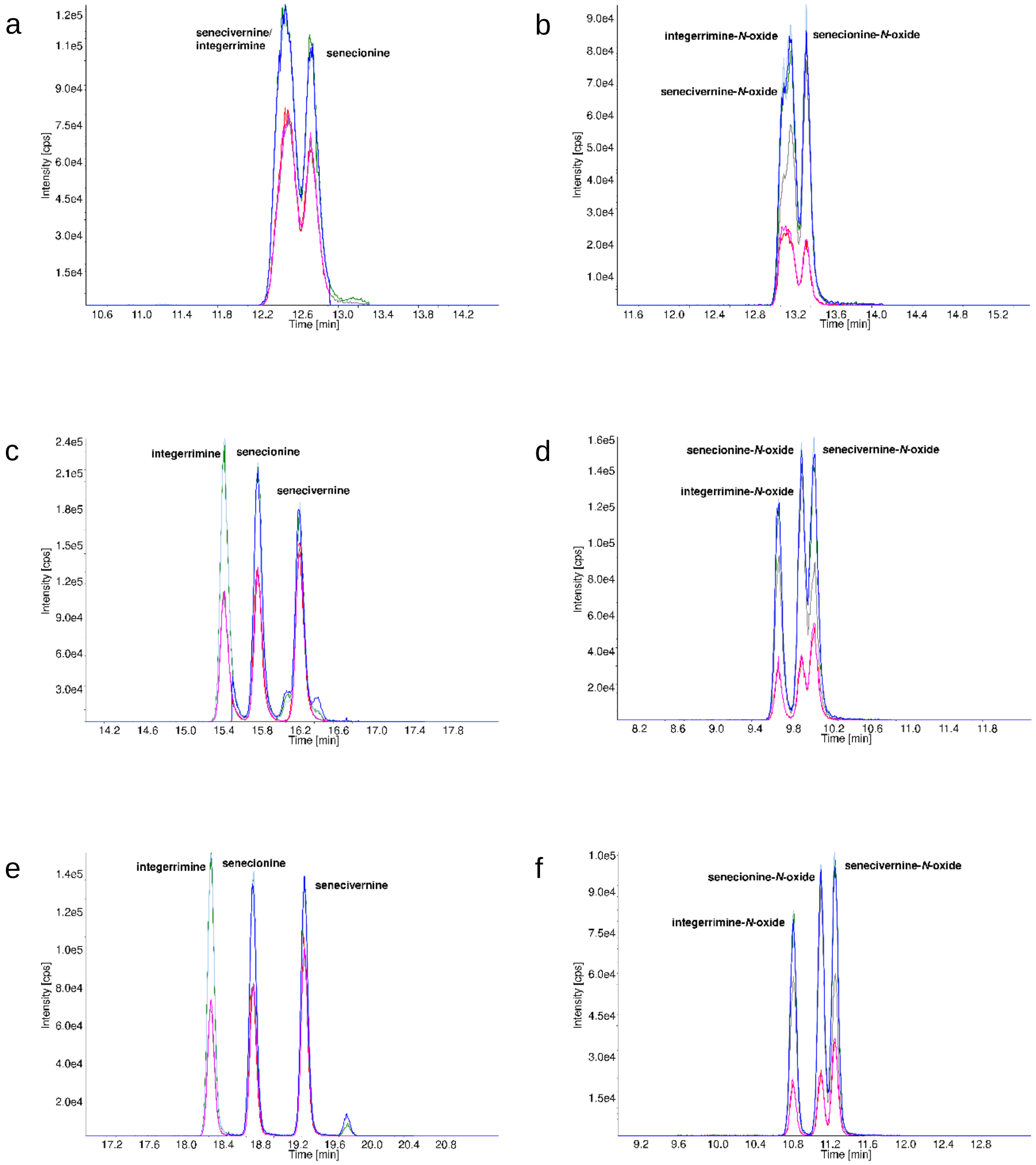

Fig. S3 Multiple reaction monitoring (MRM) chromatograms of isomeric pyrrolizidine alkaloids integerrimine, senecionine and senecivernine and their corresponding *N*-oxides under acidic solvent conditions using a 150 x 2.1 mm KinetexTM 5 µm EVO C18 column (a + b) and alkaline solvent conditions using a 150 x 2.1 mm KinetexTM 5 µm EVO C18 column (c + d) and alkaline solvent conditions using a 100 x 2.1 mm KinetexTM 2.6 µm EVO C18 (e +f). Individual mass transitions are illustrated with different colours.

Table S3 Recovery, calculated using calibration standards in methanol/water (10/90, v/v), and precision, expressed as the relative standard deviation (RSD), of sample extraction and clean-up procedures using C18 cartridges with n-hexane (n=3) and polymer cation exchange (PCX) cartridges with and without n-hexane (n=4). Replicates were spiked to a concentration of 12.3 ng/mL in the final measuring solution. For Abbreviations see **Tab. 1**

Analyte	PCX material + n-hexane		PCX material - n-hexane		C18 material + n-hexane	
	Recovery [%]	RSD [%]	Recovery [%]	RSD [%]	Recovery [%]	RSD [%]
AcIm	66.6	3.7	67.6	0.9	53.3	6.4
AcImN	0.5	11.8	0.6	25.1	55.0	7.3
AcLy	64.8	3.6	65.3	2.0	53.6	5.2
AcLyN	0.6	5.3	0.7	16.6	99.7	6.9
At	66.6	1.9	69.3	1.6	83.6	5.1
Ec	61.2	1.8	57.9	1.6	87.0	4.8
EcN	64.8	2.5	58.5	4.1	63.8	3.0
Em	68.8	4.6	71.9	0.6	76.2	15.3
EmN	72.0	3.1	70.7	1.3	72.7	5.7
En	69.9	0.9	65.1	2.1	73.2	6.9
EnN	70.7	3.1	65.4	3.6	58.4	4.3
Eu	68.9	1.8	65.6	2.3	42.4	2.7
EuN	62.1	3.7	62.8	6.5	78.6	6.3
Hs	74.3	2.1	78.5	1.1	7.4	6.1
HsN	74.4	2.7	73.9	1.7	46.9	3.7
Ht	58.9	3.0	59.3	3.1	72.4	1.8
HtN	76.7	2.2	74.5	3.5	63.4	3.0
Ic	73.7	3.2	67.0	1.9	52.7	4.5
IcN	92.0	2.6	88.8	2.8	68.5	5.2
Ig	65.4	1.9	66.2	1.6	63.7	11.9
IgN	70.1	2.2	64.0	1.1	61.3	4.1
Im	74.4	2.5	67.8	1.5	53.2	4.4
ImN	107.6	2.7	106.0	2.7	60.6	4.3
Jb	71.4	2.0	69.3	2.1	52.5	10.3
JbN	70.5	3.6	62.7	3.0	30.9	4.0
Jl	60.9	5.0	55.7	2.7	31.0	2.8
JlN	58.9	4.5	52.6	5.7	62.9	3.1
Jn	43.6	2.9	44.0	3.7	64.8	18.1
Lc	8.5	4.5	8.8	5.5	50.8	4.6
LcN	73.1	1.6	74.8	1.1	61.4	5.3
Ly	75.3	3.1	68.8	1.5	61.0	5.9
LyN	87.8	2.6	85.4	1.8	62.5	4.3
Mc	66.7	2.6	58.2	2.8	51.9	2.7
McN	57.5	5.7	51.7	5.0	69.2	1.5
Mk	49.2	2.2	50.6	2.6	67.2	9.5
MkN	7.6	7.4	8.7	29.6	48.9	62.4
Mx	52.9	3.0	48.9	1.9	53.8	7.0
MxN	126.8	2.3	106.1	3.5	40.5	4.1

Table S3 *(continued)*

Analyte	PCX material + n-hexane		PCX material - n-hexane		C18 material + n-hexane	
	Recovery [%]	RSD [%]	Recovery [%]	RSD [%]	Recovery [%]	RSD [%]
Rl	41.3	2.2	41.4	2.1	63.1	6.2
RlN	21.5	1.1	19.6	8.8	39.8	2.4
Rr	73.1	2.8	67.2	1.5	52.5	6.0
RrN	72.9	2.3	67.1	3.5	69.1	3.2
Rs	57.3	1.4	56.9	1.6	48.5	5.2
RsN	68.6	4.1	59.1	3.1	49.8	1.1
Sc	63.6	3.7	65.6	1.8	25.6	9.4
ScN	59.9	3.7	64.2	3.5	58.2	1.4
Sco	62.9	2.3	64.7	0.7	53.6	7.5
Sd	58.0	3.2	60.2	0.9	32.2	3.0
Sk	73.0	2.4	68.3	0.6	45.1	5.5
Sl	39.4	1.6	36.3	3.9	38.4	7.0
SlN	60.4	6.8	52.6	4.5	37.5	3.2
Sp	58.0	3.1	58.8	1.1	29.3	5.7
SpN	32.3	4.8	32.3	4.4	16.5	8.0
Sv	60.2	5.9	63.3	1.1	32.2	4.3
SvN	69.1	4.1	64.2	1.6	12.4	7.4
Td	59.9	2.9	59.9	2.8	17.5	5.6
Us	59.0	1.6	59.9	0.5	44.6	4.7
UsN	73.7	4.3	64.4	2.7	50.7	6.5
Minimum	0.5	0.9	0.6	0.5	7.4	1.1
Mean	61.7	3.3	59.3	3.7	53.0	6.5
Median	65.1	2.9	64.1	2.2	53.4	5.2
Maximum	126.8	11.8	106.1	29.6	99.7	62.4

a

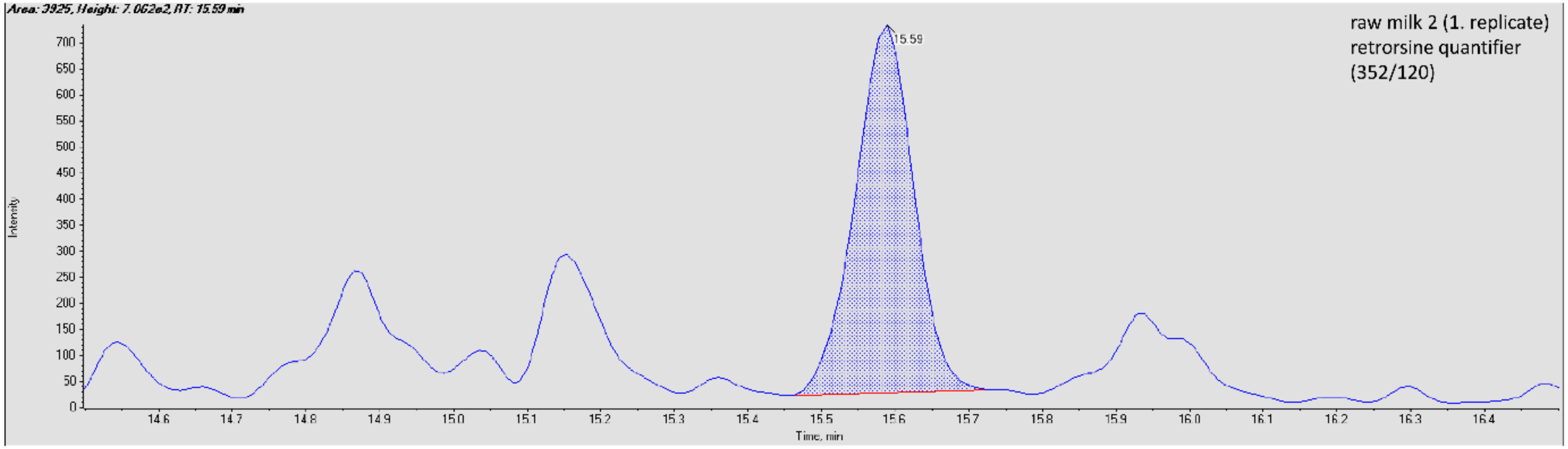

b

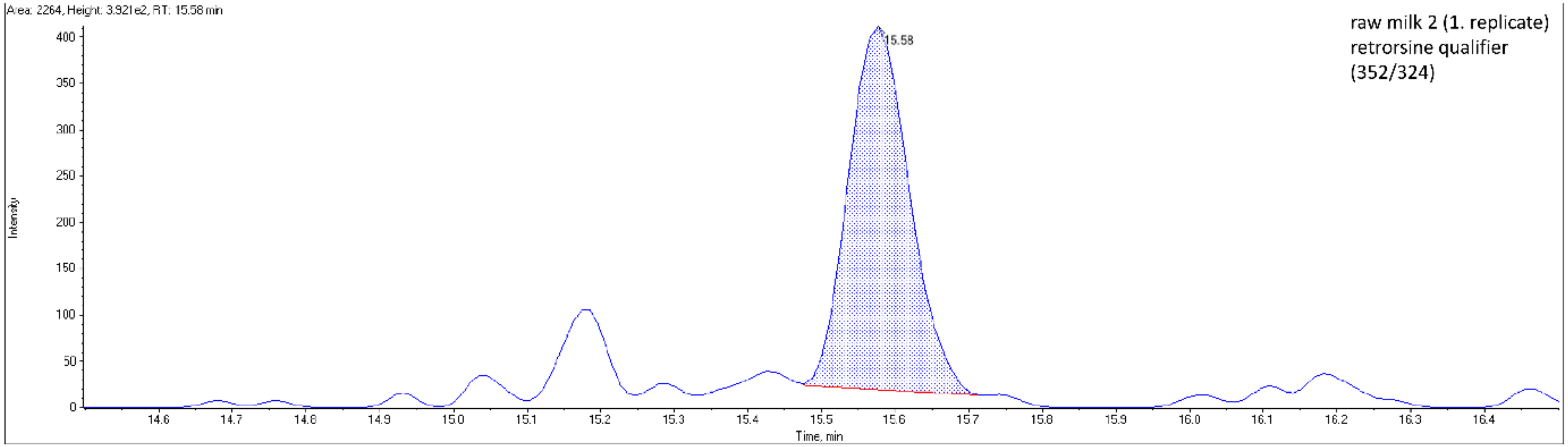

Fig. S4 Chromatograms of retrosine quantifier (a) and qualifier (b) mass transitions obtained from raw milk sample 2 (1. Replicate)

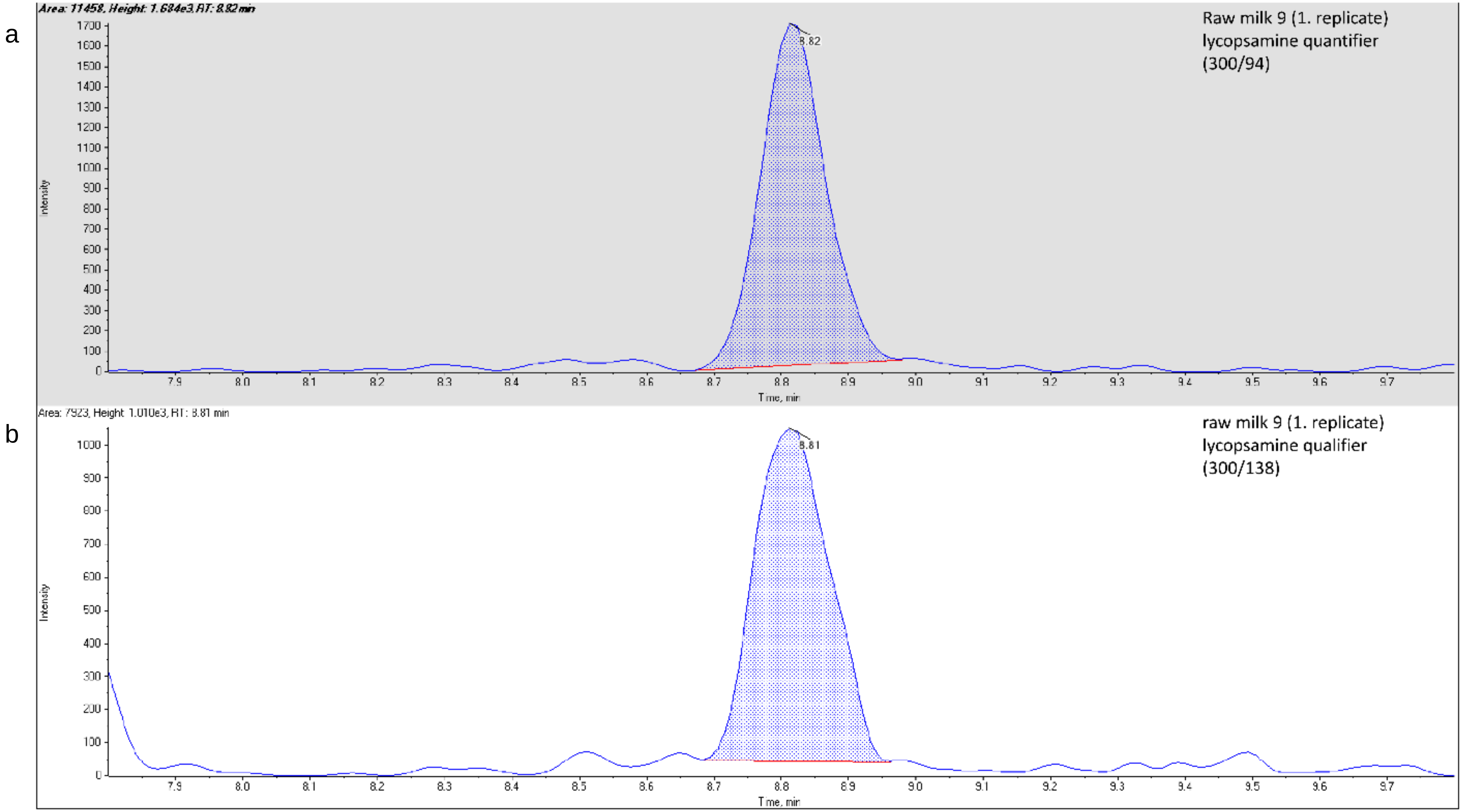

Fig. S5 Chromatograms of lycopsamine quantifier (a) and qualifier (b) mass transitions obtained from raw milk sample 9 (1. Replicate)

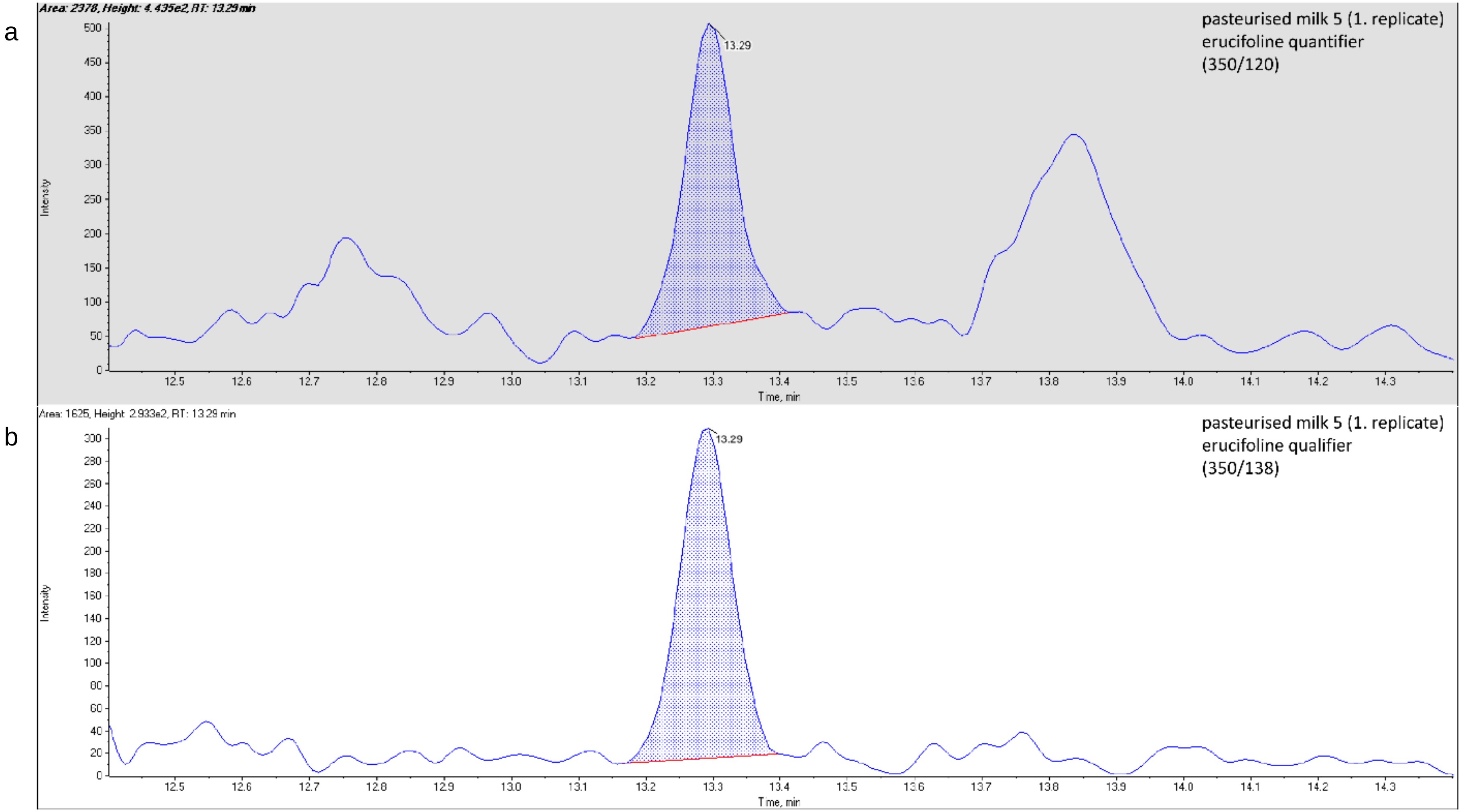

Fig. S6 Chromatograms of erucifoline quantifier (a) and qualifier (b) mass transitions obtained from pasteurised milk sample 3 (1. Replicate)

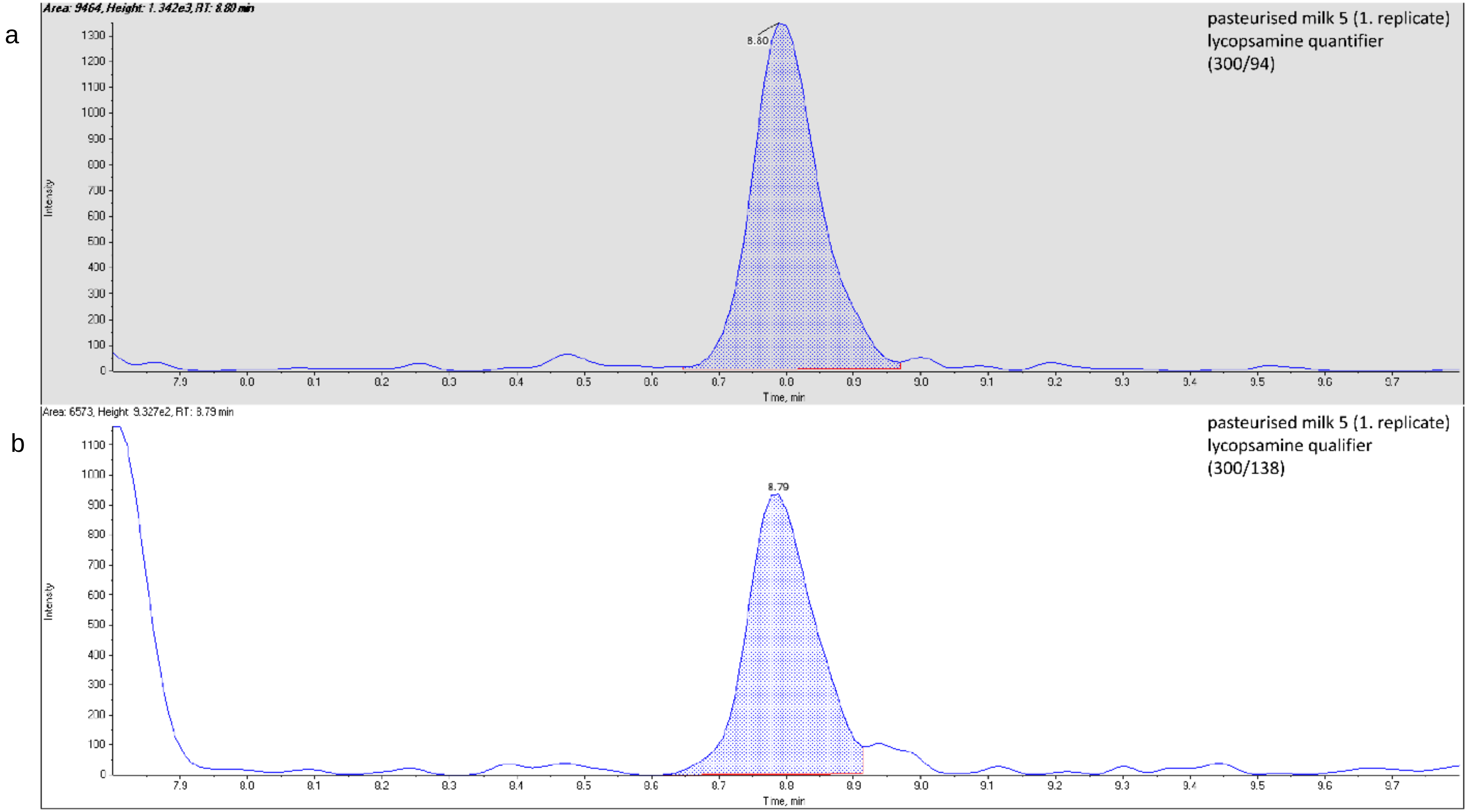

Fig. S7 Chromatograms of lycopsamine quantifier (a) and qualifier (b) mass transitions obtained from pasteurised milk sample 3 (1. Replicate)

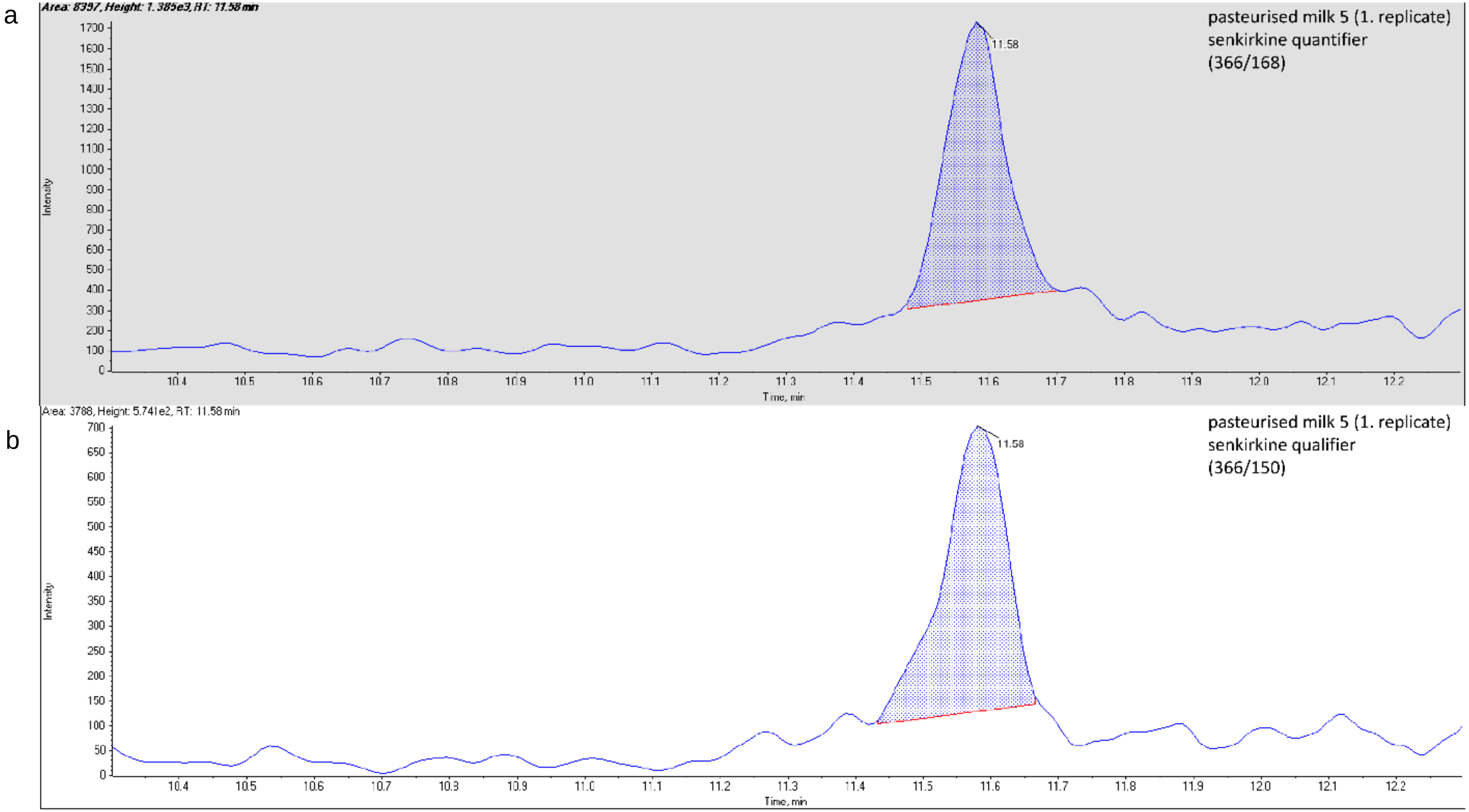

Fig. S8 Chromatograms of senkirkine quantifier (a) and qualifier (b) mass transitions obtained from pasteurised milk sample 3 (1. Replicate)

Table S4 Results of the confirmation analysis. Calculated content in each spiked replicate and theoretical spiked pyrrolizidine alkaloid amount.

Analyte	Calculated Concentration [µg/L]			Spiked Amount [µg/L]
	Replicate 1	Replicate 2	Replicate 3	
Erucifoline	0.010	0.008[a]	0.011	0.010
Lycopsamine	0.017	0.016	0.016	0.015
Retrorsine	0.015	n. d.	0.017	0.020
Senkirkine	0.014	0.018	0.019	0.020

n.d., not detected
[a] calculated value below the LOD

References

1. Hoogenboom LAP, Mulder PPJ, Zeilmaker MJ, van den Top HJ, Remmelink GJ, Brandon EFA, et al. Carry-over of pyrrolizidine alkaloids from feed to milk in dairy cows. Food Addit Contam Part A Chem Anal Control Expo Risk Assess. 2011;28(3):359-72.
2. Griffin C. Investigation of Pyrrolizidine Alkaloids in Foods using Liquid Chromatography Mass Spectrometry. *PhDs* [online]. 2014. Available from: https://sword.cit.ie/scidiss/1 [last accessed: 14 July 2022]
3. Yoon SH, Kim MS, Kim SH, Park HM, Pyo H, Lee YM, et al. Effective application of freezing lipid precipitation and SCX-SPE for determination of pyrrolizidine alkaloids in high lipid foodstuffs by LC-ESI-MS/MS. J Chromatogr B Analyt Technol Biomed Life Sci. 2015;992:56-66.
4. Huybrechts B, Callebaut A. Pyrrolizidine alkaloids in food and feed on the Belgian market. Food Addit Contam Part A Chem Anal Control Expo Risk Assess. 2015;32(11):1939-51.
5. Mulder PPJLS, P.; These, A.; Preiss-Weigert, A.; Castellari, M. Occurrence of Pyrrolizidine Alkaloids in food. EFSA supporting publication. 2015:EN-859116 pp.
6. Mulder PPJ, Klijnstra MD, Goselink RMA, van Vuuren AM, Cone JW, Stoopen G, et al. Transfer of pyrrolizidine alkaloids from ragwort, common groundsel and viper's bugloss to milk from dairy cows. Food Addit Contam Part A Chem Anal Control Expo Risk Assess. 2020;37(11):1906-21.
7. De Nijs M, Mulder PPJ, Klijnstra MD, Driehuis F, Hoogenboom RLAP. Fate of pyrrolizidine alkaloids during processing of milk of cows treated with ragwort. Food Addit Contam Part A Chem Anal Control Expo Risk Assess. 2017;34(12):2212-9.
8. Chung SWC, Lam ACH. Investigation of pyrrolizidine alkaloids including their respective N-oxides in selected food products available in Hong Kong by liquid chromatography electrospray ionisation mass spectrometry. Food Addit Contam Part A Chem Anal Control Expo Risk Assess. 2017;34(7):1184-92.
9. Chen Y, Li L, Xiong F, Xie Y, Xiong A, Wang Z, et al. Rapid identification and determination of pyrrolizidine alkaloids in herbal and food samples via direct analysis in real-time mass spectrometry. Food Chem. 2021;334:127472.

10. Zheng W, Yoo K-H, Choi J-M, Park D-H, Kim S-K, Kang Y-S, et al. A modified QuEChERS method coupled with liquid chromatography-tandem mass spectrometry for the simultaneous detection and quantification of scopolamine, L-hyoscyamine, and sparteine residues in animal-derived food products. J Adv Res. 2019;15:95-102.

11. Lamp J, Knappstein K, Walte HG, Krause T, Steinberg P, Schwake-Anduschus C. Transfer of tropane alkaloids (atropine and scopolamine) into the milk of subclinically exposed dairy cows. Food Control. 2021;126.
